# Sex differences in the associations of multidomain risk factor profiles with incident major adverse cardiovascular events

**DOI:** 10.3389/fcvm.2026.1911138

**Published:** 2026-09-03

**Authors:** Yuhui Yang, Xi Chen, Yuhao Hu, Xiaochen Wang, Huili Wang, Youquan Wei, Feilong Zhang, Zhen Dong, Ronghui Yu

**Affiliations:** 1 Department of Cardiology, National Cardiovascular Disease Regional Center for Anhui, The First Affiliated Hospital of Anhui Medical University, Hefei, Anhui, China; 2 Department of Cardiology, The Second Affiliated Hospital of Anhui Medical University, Hefei, Anhui, China; 3 Department of Cardiology, Lvliang First People's Hospital, Lvliang Hospital of Shanxi Medical University, Lvliang, Shanxi, China; 4 Department of Cardiology, The First Affiliated Hospital of Wannan Medical University (Yijishan Hospital of Wannan Medical University), Wuhu, Anhui, China; 5 Department of Cardiology, No.2 People's Hospital of Fuyang City, Fuyang Infectious Disease Clinical College of Anhui Medical University, Fuyang, Anhui, China

**Keywords:** major adverse cardiovascular events (MACE), polygenic risk score (PRS), risk factor profiles, sex differences, UK biobank

## Abstract

**Background:**

Major adverse cardiovascular events (MACE) exhibit sex-specific disparities, but comprehensive analyses of combined genetic and clinical risk factor profiles across sexes remain limited.

**Methods:**

This prospective cohort study included 397,806 UK Biobank participants (55.1% female) without baseline MACE. Five weighted risk scores [social-demographic, lifestyle, metabolic, clinical comorbidity, polygenic risk score (PRS)] were evaluated for associations with incident MACE via sex-stratified Cox proportional hazards regression, interaction analyses, and population-attributable risk (PAR) assessments.

**Results:**

Over a median follow-up of 15.6 years, 32,019 participants (8.0%) developed MACE (11.1% males vs. 5.5% females). Social-demographic (Interaction effect: HR 0.91, 95% CI 0.89–0.93), lifestyle (Interaction effect: HR 0.94, 95% CI 0.91–0.97), and clinical comorbidity (Interaction effect: HR 0.85, 95% CI 0.82–0.87) scores exhibited stronger MACE associations in females than males, while males had higher PRS-related risk (Interaction effect: HR 1.07, 95% CI 1.03–1.11, *P* for interaction < 0.001). Pairwise interaction patterns differed by sex. PRS integration improved MACE prediction in males (*Δ*C-statistic = 0.010, *P* < 0.001) but not in females (*Δ*C-statistic = 0.005, *P* = 0.299). PAR ranking differed: while social-demographic and clinical comorbidity scores remained the top two contributors across sexes, polygenic risk emerged as the third largest contributor in males (15.9%), and unhealthy lifestyle was the third largest in females (15.6%).

**Conclusions:**

Sex-specific differences in the effects and contributions of risk factor profiles highlight the need for tailored MACE prevention strategies. Polygenic risk provided modest additional discrimination in males, supporting further evaluation of precise cardiovascular risk assessment.

## Introduction

Cardiovascular disease (CVD), particularly major adverse cardiovascular events (MACE), remains the leading cause of death globally and imposes a substantial economic burden on both individuals and society (1–4). Early identification of individuals at high risk of MACE is critical (5). Population-based epidemiological studies have identified several clinical risk factors for MACE (1, 6–11). The SCORE2 clinical prediction model, incorporating just seven common risk factors, offers notable practical advantages for MACE prediction, with high feasibility and scalability (12). However, the model is constrained by a lack of comprehensive variables, potentially failing to capture risk heterogeneity.

Advances in genome-wide association studies (GWAS) have uncovered significant genetic associations with CVD (13–16). While the predictive performance of polygenic risk score (PRS) for CVD has yielded mixed results (17, 18) several studies have demonstrated that integrating PRSs into established models or risk factor panels substantially improves predictive accuracy and facilitates precise MACE risk stratification but did not address sex differences (19–21). Moreover, few studies have indicated that interactions between sex and risk factors, as well as interplays among risk factors themselves, exert variable impacts on MACE risk (22–24). Although existing evidence suggests sex-specific genetic architectures and effects in CVD (25–28), sex differences in the integration of genetic risk into clinical factors for predicting incident MACE remain incompletely characterized. Few studies have integrated conventional and polygenic risk factors within a unified multidomain framework to compare their associations, interactions, predictive contributions, and population-level burdens between females and males.

Thus, using the prospective UK Biobank cohort, we aimed to systematically evaluate sex differences in the associations, interactions, predictive contributions, and population-attributable burdens of five interconnected risk domains (social-demographic characteristics, lifestyles, metabolic indicators, clinical comorbidities, and polygenic risk) with incident MACE.

## Methods

### Study population

This prospective study used data from the UK Biobank, a longitudinal cohort of over 500,000 volunteers recruited between 2006 and 2010. Details of the study design and data collection have been reported previously (29, 30). Initially, 502,112 participants were included. We excluded 19,445 participants with a baseline history of MACE, 1,235 who withdrew from the study, 15,476 with unavailable PRS, and 68,150 with missing clinical risk factor data, yielding a final analytical cohort of 397,806 participants (Supplementary Figure S1). This study was conducted under an approved UK Biobank application (application number: 280417). All participants provided written informed consent. Ethical approval was obtained from the North West Research Ethics Committee (11/NW/0382, 21/NW/0157) and the Ethics Committee of The First Affiliated Hospital of Anhui Medical University (PJ 2024-13-46).

### Ascertainment of candidate risk factors

Genetic risk was assessed using standard PRS from the UK Biobank, including those for atrial fibrillation (AF PRS), cardiovascular disease (CVD PRS), coronary artery disease (CAD PRS), and stroke (Stroke PRS). Detailed methods have been described previously (31–33). High polygenic risk was defined as the highest quintile of PRS.

Comprehensive established and potential MACE risk factor profiles were assessed, including social-demographic characteristics, lifestyles, metabolic factors, and clinical comorbidities (Supplementary Appendix S1). Definitions of all risk factor profiles are detailed in Supplementary Table S1. Missingness was summarized overall and by sex, and baseline characteristics were directly compared between participants included in the complete-case analysis and those excluded because of missing clinical risk-factor data, with standardized mean differences (SMD) used to quantify between-group differences (Supplementary Tables S2 and S3). Overweight/obesity was defined as a body mass index (BMI) ≥ 25 kg/m^2^; all other continuous clinical risk factor profiles were categorized using optimal cutoffs determined by the maximum Youden index in the overall cohort (Supplementary Table S4). The same cutoffs were applied to females and males.

### Outcomes

The primary outcome was the first occurrence of MACE, including heart failure, myocardial infarction, stroke, and cardiovascular mortality. Outcome was ascertained via self-reported medical conditions, death registries, and the first event documented in primary care visits or hospital admissions, using International Classification of Diseases, Tenth Revision (ICD-10), code (Supplementary Table S1). Follow-up was censored at the first occurrence of MACE, documented death, or the study end date (October 25, 2024).

### Statistical analysis

Baseline characteristics are presented as means ± standard deviations (SD) for continuous variables and counts (percentages) for categorical variables. Significant predictors of incident MACE were identified using least absolute shrinkage and selection operator (LASSO) Cox regression (Supplementary Figure S2). The LASSO variable-selection procedure and subsequent estimation of regression coefficients for risk-score weighting were performed using the overall analytical cohort. Multicollinearity in the Cox proportional hazards models was assessed using generalized variance inflation factors (GVIF) and no substantial multicollinearity was detected (Supplementary Table S5). Pairwise correlations among the four continuous PRSs were additionally assessed using Pearson correlation coefficients (Supplementary Figure S3).

Weighted risk scores for the five domains were constructed using estimates derived from the overall cohort. Individual risk factors were weighted according to *β* coefficients estimated from multivariable Cox regression models for incident MACE, and the weighted components were summed to generate domain-specific scores (34). For the polygenic risk domain, AF PRS, CVD PRS, CAD PRS, and Stroke PRS were dichotomized according to the top quintile of their distributions and simultaneously entered into the multivariable Cox model to derive mutually adjusted β coefficients for weighting. Age was included in the social-demographic risk domain as a binary variable (≥ 60 years), consistent with the categorical scoring framework applied to other risk factors. Included risk factors are detailed in Supplementary Appendix S1. Cox proportional hazards models examined associations between the risk factor profiles and incident MACE in the overall and sex-stratified populations. Multiplicative interaction terms were included to assess sex-specific associations.

Robustness was verified via several sensitivity analyses: 1) Excluding MACE cases occurring within the first 2 years of follow-up (*n* = 3,528); 2) Excluding self-reported MACE cases (*n* = 848); 3) Excluding non-White participants (*n* = 18,007); 4) Competing risk analysis for non-cardiovascular mortality using Fine-Gray sub-distribution hazards models; 5) Using multiple imputation by random forest to impute baseline missing data for clinical risk factor profiles (Supplementary Table S2) and mitigate bias from complete case analysis; 6) Using an alternative conventional MACE definition excluding heart failure, defined as a composite outcome of myocardial infarction, stroke, and cardiovascular mortality. Component-wise analyses were additionally performed for heart failure, myocardial infarction, stroke, and cardiovascular mortality, with sex interactions evaluated separately for each domain-specific risk score. To assess domain overlap, we reconstructed the metabolic risk score after excluding HbA1c while retaining diabetes mellitus in the clinical comorbidity domain and repeated association, interaction, and PAR analyses. Furthermore, we performed a sensitivity analysis in which continuous risk factors were modeled using restricted cubic splines with four knots, including interactions between sex and the complete spline basis, while adjusting for the remaining covariates as in the primary analysis, to evaluate whether the observed sex-specific associations persisted under continuous nonlinear modeling.

Kaplan–Meier cumulative incidence curves were used to estimate the absolute risk of MACE in the overall and sex-stratified populations. Cox proportional hazards models including all pairwise interactions among the five categorized domain scores were constructed to assess associations with MACE risk and explore sex-specific differences.

Stepwise incremental value in MACE prediction was assessed by Harrell's C-statistic and validated via 5-fold cross-validation. In addition, bootstrap optimism correction with 1,000 resamples was performed to assess potential optimism in model performance. The LASSO variable-selection procedure and estimation of the *β* coefficients used for risk-score weighting were not repeated within each bootstrap resample. The difference in incremental C-statistic after adding the polygenic risk domain between females and males was additionally assessed using bootstrap resampling. Net reclassification improvement (NRI) and integrated discrimination improvement (IDI) were used to quantify improvements in predictive performance. Decision curve analysis (DCA) evaluated clinical net benefit. Ten-year calibration was evaluated using 5-fold cross-validated calibration plots and Brier scores. All aforementioned analyses included sex-stratified subsets to explore sex-specific differences.

The relative importance and population-level impact of individual risk factor profiles and weighted domain scores were evaluated using population-attributable risk (PAR) percentages for MACE in the overall and sex-stratified populations. PAR percentages were estimated as descriptive measures of the population-level burden associated with each risk factor profile (35).

Because PAR estimates quantify population-level burden rather than mutually exclusive contributions, estimates across domains should not be summed. Statistical significance was defined as a two-tailed *P* < 0.05. For sex-interaction analyses, Bonferroni correction was applied according to the number of interaction tests within each analysis. In the primary analysis, which included 28 interaction tests involving the individual risk factors and domain-specific risk scores, a Bonferroni-adjusted threshold of *P* for interaction < 0.002 (0.05/28) was applied. In sensitivity and secondary analyses restricted to the five domain-specific risk scores, a Bonferroni-adjusted threshold of *P* for interaction < 0.01 (0.05/5) was applied within each analysis or outcome. The proportional hazards assumption was tested using Schoenfeld residuals. All analyses were performed in R (version 4.4.0) on the UK Biobank Research Analysis Platform.

## Results

### Baseline characteristics

This study included 397,806 participants (55.1% female) with a mean age of 56.3 ± 8.1 years. Baseline characteristics stratified by sex are presented in Table 1. Over a median follow-up of 15.6 years, 32,019 participants (8.0%) experienced incident MACE. The cumulative incidence of MACE was significantly higher in males than in females (11.1% vs. 5.5%; *P* < 0.001).

**Table 1:** Baseline characteristics.

Characteristics	All participants *N* = 397,806	Females *N* = 219,273	Males *N* = 178,533	*P* value
Social-demographic characteristics				
Age ≥ 60	166,816 (41.9%)	89,907 (41.0%)	76,909 (43.1%)	**< 0.001**
Ethnicity (White)	379,799 (95.5%)	209,531 (95.6%)	170,268 (95.4%)	**0.005**
Higher TDI	90,753 (22.8%)	49,523 (22.6%)	41,230 (23.1%)	**< 0.001**
Less education	263,660 (66.3%)	148,434 (67.7%)	115,226 (64.5%)	**< 0.001**
Lifestyle behaviours				
Current smoker	40,660 (10.2%)	19,157 (8.7%)	21,503 (12.0%)	**< 0.001**
Excessive alcohol intake	72,418 (18.2%)	18,448 (8.4%)	53,970 (30.2%)	**< 0.001**
Longer sleep duration	29,096 (7.3%)	16,968 (7.7%)	12,128 (6.8%)	**< 0.001**
Sedentary	180,246 (45.3%)	84,077 (38.3%)	96,169 (53.9%)	**< 0.001**
Metabolic indicators				
Overweight/obesity	262,647 (66.0%)	130,232 (59.4%)	132,415 (74.2%)	**< 0.001**
Higher LDL	62,405 (15.7%)	31,218 (14.2%)	31,187 (17.5%)	**< 0.001**
Higher TG	194,057 (48.8%)	88,833 (40.5%)	105,224 (58.9%)	**< 0.001**
Higher HbA1c	134,492 (33.8%)	73,001 (33.3%)	61,491 (34.4%)	**< 0.001**
Lower IGF-1	169,139 (42.5%)	101,848 (46.4%)	67,291 (37.7%)	**< 0.001**
Clinical comorbidities				
DM	17,859 (4.5%)	6,934 (3.2%)	10,925 (6.1%)	**< 0.001**
Hypertension	104,294 (26.2%)	50,473 (23.0%)	53,821 (30.1%)	**< 0.001**
AF	4,995 (1.3%)	1,637 (0.7%)	3,358 (1.9%)	**< 0.001**
VHD	2,178 (0.5%)	1,118 (0.5%)	1,060 (0.6%)	**< 0.001**
Cardiomyopathy	396 (0.1%)	120 (0.1%)	276 (0.2%)	**< 0.001**
VTE	11,013 (2.8%)	6,770 (3.1%)	4,243 (2.4%)	**< 0.001**
Hyperthyroidism	4,271 (1.1%)	3,538 (1.6%)	733 (0.4%)	**< 0.001**
Dyslipidemia	52,493 (13.2%)	23,253 (10.6%)	29,240 (16.4%)	**< 0.001**
COPD	6,748 (1.7%)	3,347 (1.5%)	3,401 (1.9%)	**< 0.001**
CKD	4,035 (1.0%)	2,226 (1.0%)	1,809 (1.0%)	0.965
OSA	1,700 (0.4%)	421 (0.2%)	1,279 (0.7%)	**< 0.001**
Polygenic risk scores				
High AF PRS	79,561 (20.0%)	44,152 (20.1%)	35,409 (19.8%)	**0.010**
High CVD PRS	79,561 (20.0%)	44,698 (20.4%)	34,863 (19.5%)	**< 0.001**
High CAD PRS	79,561 (20.0%)	44,531 (20.3%)	35,030 (19.6%)	**< 0.001**
High Stroke PRS	79,561 (20.0%)	44,148 (20.1%)	35,413 (19.8%)	**0.019**
Outcome				
Incident MACE	32,019 (8.0%)	12,131 (5.5%)	19,888 (11.1%)	**< 0.001**

Data are presented as *n* (%). TDI, Townsend deprivation index; LDL, low-density lipoprotein; TG, triglycerides; HbA1c, glycated hemoglobin; IGF-1, insulin-like growth factor 1; DM, diabetes mellitus; AF, atrial fibrillation; VHD, valvular heart disease; VTE, venous thromboembolism; COPD, chronic obstructive pulmonary disease; CKD, chronic kidney disease; OSA, obstructive sleep apnea; PRS, polygenic risk scores; CVD, cardiovascular disease; and CAD, coronary artery disease.

Bold values indicate *P* < 0.05.

### Comprehensive risk scores and MACE in the overall and sex-stratified populations

**Figure 1:**
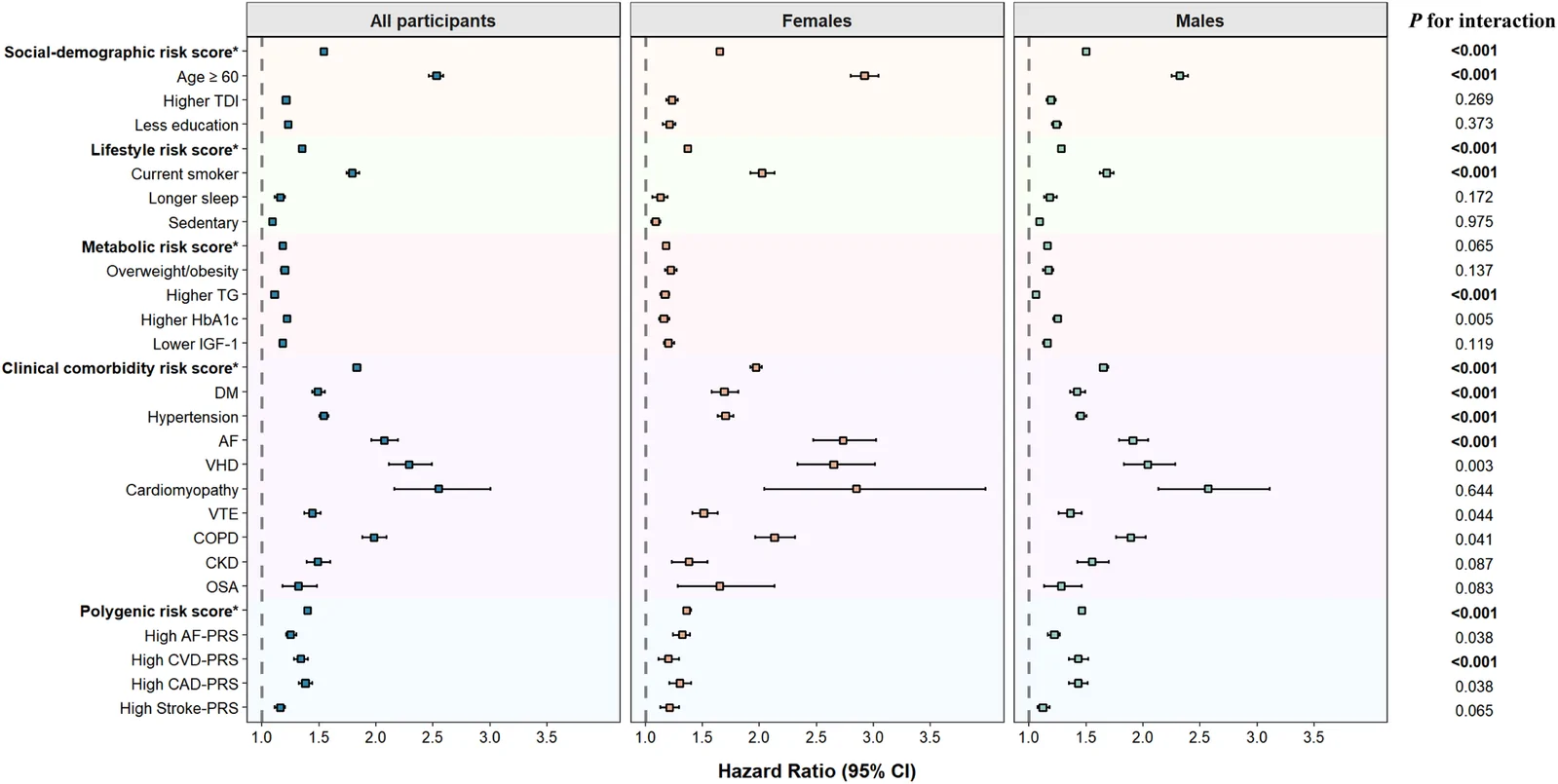
Multivariable-Adjusted hazard ratios of incident MACE associated with risk factor profiles in the overall and Sex-stratified populations. *HR indicates the risk of incident MACE associated with a per 1-point increase. Models were mutually adjusted for individual risk factor profiles, with sex additionally adjusted in all participants. Statistical significance was defined using Bonferroni-corrected thresholds of *P* for interaction < 0.002 (0.05/28).

In the multivariable model, all selected predictors remained independently associated with incident MACE (Supplementary Table S6), with event counts underlying the estimates for individual clinical comorbidities provided in Supplementary Table S7. All five domain-specific risk scores demonstrated a significant association with MACE, with each 1-point increase corresponding to an 18% to 83% higher risk (all *P* < 0.001, Figure 1).

Significant sex differences were observed in the associations of the weighted risk scores and individual risk factors with incident MACE (Figure 1, Supplementary Figure S4 and Table S8). Higher social-demographic risk score (HR: 1.65 vs. 1.50, *P* for interaction < 0.001), lifestyle risk score (HR: 1.37 vs. 1.28, *P* for interaction < 0.001), and clinical comorbidity risk score (HR: 1.97 vs. 1.65, *P* for interaction < 0.001) showed stronger associations with MACE in females than in males. In contrast, males exhibited a higher polygenic risk-related MACE risk (HR: 1.46 vs. 1.36, *P* for interaction < 0.001). Sensitivity analyses, including exclusion of MACE cases within the first 2 years of follow-up, exclusion of cases ascertained by self-report only, exclusion of non-White participants, competing risk analysis for non-cardiovascular mortality, multiple imputation for missing clinical risk factors, and use of an alternative MACE definition excluding heart failure (Supplementary Tables S9–S14), yielded broadly consistent results. In addition, reconstruction of the metabolic risk score after excluding HbA1c to address potential overlap between metabolic and clinical comorbidity domains yielded similar findings (Supplementary Table S15). Component-wise analyses showed consistently stronger associations of the clinical comorbidity score in females across all four outcomes, whereas sex differences in the other risk domains varied by outcome; the polygenic risk score was more strongly associated with myocardial infarction in males (*P* for interaction = 0.006; Supplementary Table S16). Kaplan–Meier cumulative incidence curves for MACE according to categorized risk scores are presented in Supplementary Figures S5 and S6.

Pairwise interactions among the five categorized risk scores were further explored on the multiplicative scale (Supplementary Figure S7). Interaction patterns differed by sex. In females, significant interactions mainly involved social-demographic risk and conventional risk domains, with an additional interaction between metabolic and clinical comorbidity risks. In males, polygenic risk additionally interacted with social-demographic and clinical comorbidity risks. These findings suggested sex-specific heterogeneity in the joint associations of multidomain risk profiles with incident MACE.

### Sex-specific incremental value of stepwise combinations of risk scores

**Figure 2:**
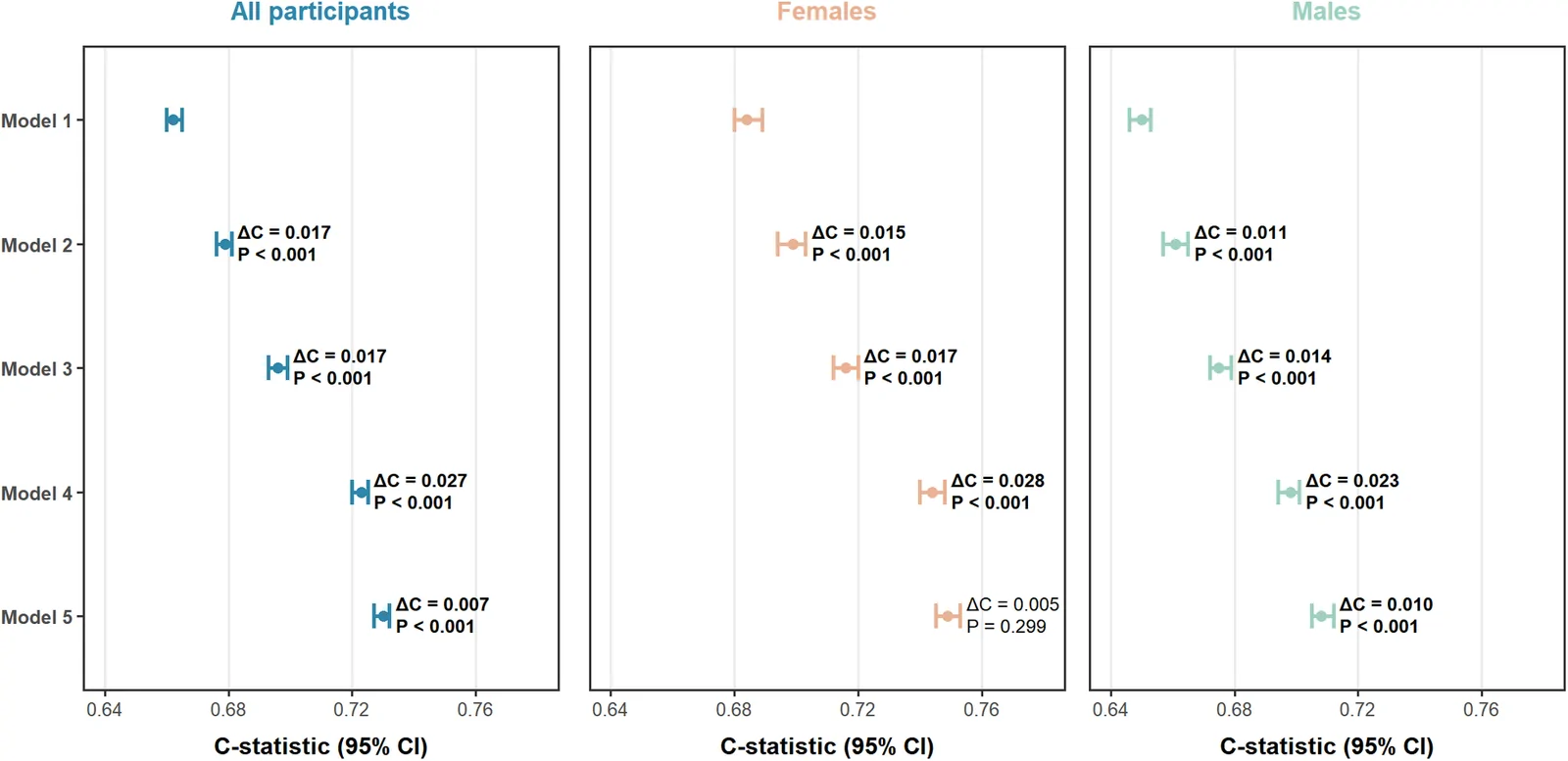
Incremental predictive value for MACE risk with stepwise combinations of the risk scores in the overall and sex-stratified populations. Incremental predictive value for MACE risk with stepwise combinations of the five risk scores in all participants, females, and males. *Δ*C indicates the changes in Harrell's C-statistic relative to the preceding model. Model 1: social-demographic risk score; Model 2: Model 1 + lifestyle risk score; Model 3: Model 2 + metabolic risk score; Model 4: Model 3 + clinical comorbidity risk score; Model 5: Model 4 + polygenic risk score. *P* values were corrected by Bonferroni (*n* = 4).

Sequential addition of lifestyle, metabolic, clinical comorbidity, and polygenic risk scores to the social-demographic risk score improved model discrimination in the overall and male populations (all Bonferroni-corrected *P* < 0.001; Figure 2). Positive NRI and IDI values were also observed across the overall and sex-stratified populations (all *P* < 0.001; Supplementary Table S17). The addition of the polygenic risk domain resulted in a *Δ*C-statistic of 0.010 (*P* < 0.001) in males and 0.005 (*P* = 0.299) in females (Figure 2). Direct comparison using bootstrap resampling demonstrated a greater incremental improvement in males than females (difference in *Δ*C-statistic = 0.005; 95% CI, 0.004–0.007; *P* < 0.001). Bootstrap optimism-corrected analyses showed similar incremental discrimination after adding the polygenic risk domain, with corrected *Δ*C-statistics of 0.0073, 0.0052, and 0.0107 in the overall population, females, and males, respectively. Results remained robust following 5-fold cross-validation of the C-statistic (Supplementary Figure S8). DCA showed modest separation between the sequential models (Supplementary Figure S9). Calibration analyses showed close agreement between predicted and observed 10-year MACE risks before and after incorporation of the polygenic risk score in the overall and sex-stratified populations, while Brier scores decreased only minimally (Supplementary Figure S10).

Among the individual PRSs, CAD PRS produced the largest incremental improvement when entered first in the sequential analysis in the overall population (C-statistic: 0.723–0.729, *P* = 0.002) and males (C-statistic: 0.698–0.707, *P* < 0.001; Supplementary Table S18), whereas further addition of AF PRS, Stroke PRS, and CVD PRS did not significantly improve discrimination (all *P* > 0.05; Supplementary Table S18).

### Sex-Specific population-attributable risk of risk factor profiles and scores

**Figure 3:**
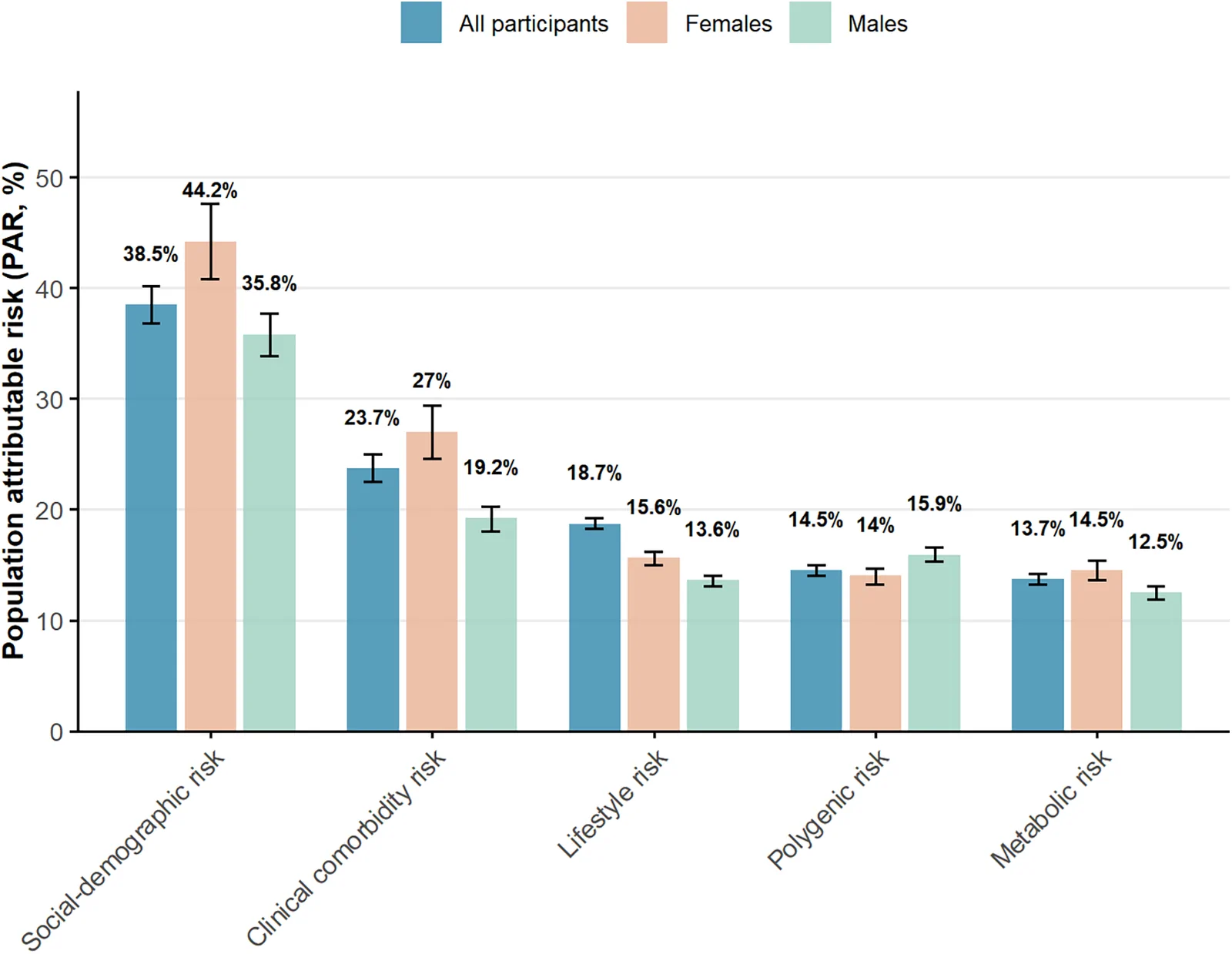
Population-attributable risk for incident MACE of risk scores in the overall and sex-stratified populations. PAR estimates represent overlapping population-level burdens associated with individual risk domains and should not be summed or interpreted as proportional contributions to cumulative MACE incidence or model performance. Models were mutually adjusted for individual risk factor scores, with sex additionally adjusted in all participants.

PAR analysis estimated the population-level burden associated with MACE-related risk factor profiles and characterized sex differences (Figure 3 and Supplementary Tables S19–S22). Among all participants, social-demographic characteristics accounted for the largest estimated PAR (38.5%; 95% CI: 36.8–40.2), followed by clinical comorbidities (23.7%; 22.5–25.0), lifestyle factors (18.7%; 18.3–19.2), polygenic risk (14.5%; 14.0–15.0), and metabolic factors (13.7%; 13.2–14.2; Figure 3). In females, the top three risk score categories remained in the same order, with social-demographic characteristics exhibiting the highest PAR (44.2%; 40.8–47.6). A notable distinction was that the metabolic factors (14.5%; 13.6–15.4) contributed more to MACE risk than polygenic risk (14.0%; 13.2–14.7). In males, the PAR of social-demographic characteristics was lower (35.8%; 33.9–37.7), and polygenic risks (15.9%; 15.3–16.6) emerged as the third leading contributor, exceeding lifestyle-related risk (13.6%; 13.1–14.0). Sensitivity analysis excluding HbA1c from the metabolic risk score yielded comparable sex-specific PAR patterns, with metabolic risk contributing more in females and polygenic risk showing a higher relative contribution in males (Supplementary Table S23). These findings indicated sex differences in the relative population-level burden associated with the five risk domains.

## Discussion

Using the prospective UK Biobank cohort, our study extends previous work by characterizing sex differences across five interconnected risk domains within a unified framework encompassing associations, pairwise interactions, incremental predictive value, and population-attributable burden. This approach revealed a broader sex-specific risk architecture rather than isolated differences in individual predictors. Key findings included sex differences in the associations of multidomain risk profiles with MACE, heterogeneous patterns of pairwise interactions among risk domains, and sex-specific differences in the incremental information provided by polygenic risk. Addition of the PRS modestly improved discrimination in males but not in females. The PAR ranking of the five risk scores varied by sex: while social-demographic and clinical comorbidity scores remained the top two contributors across sexes, polygenic risk emerged as the third largest in males and unhealthy lifestyle in females.

Previous studies have predominantly focused on individual or limited risk factors for MACE (1, 6–11, 36), hindering precise risk stratification. The incidence of MACE was higher in males than in females (11.1% vs. 5.5%), consistent with prior reports (37). However, several conventional risk domains showed stronger relative associations with MACE in females. Qin et al. demonstrated that obesity, smoking, and hypertension confer greater HF risk in females, while other studies have linked AF, diabetes mellitus (DM), and smoking to more severe MACE outcomes in females (38). Wang et al. identified hypertension as the strongest modifiable MACE risk factor associated, followed by DM (39), while a population-based longitudinal study reported no sex differences in the MACE risk associated with the two conditions (40). In our cohort, clinical comorbidities represented the highest absolute MACE risk, with hypertension and DM representing important contributors, which is consistent with select prior work (41–43).

Modifiable risk factor management yields substantial cardiovascular benefits, with each additional controlled risk factor reducing CVD risk by 20% in females and 15% in males (44). Healthy lifestyles, such as regular physical activity, non-smoking, and BMI control, are particularly protective in females (36, 45). Consistent with our results, socioeconomic status shows stronger inverse associations with CVD in females than in males (46, 47). Although sex-specific genetic architecture and sex chromosome-related mechanisms may contribute to cardiovascular heterogeneity (25, 26, 28, 48–50), the present observational analysis was not designed to determine the biological mechanisms underlying these differences.

Beyond the associations of individual risk domains, pairwise interaction patterns also differed by sex. Previous studies have reported that the associations between lifestyle and socioeconomic factors, as well as between smoking and diabetes, may not be fully independent (51, 52). In our study, interaction patterns differed between females and males: interactions in females were mainly observed among conventional risk domains, whereas males additionally showed polygenic risk-related interactions. These findings indicate sex-specific heterogeneity in the joint associations of multidomain risk profiles with MACE. As the interaction terms were evaluated on the multiplicative scale, they should be interpreted as exploratory statistical findings rather than evidence of biological synergy or causal effects.

The incremental value provided by the five risk domains also differed by sex. The SCORE2 model, validated across 70 cohorts, demonstrates moderate-to-good MACE predictive performance with sex-specific adjustments (12). And previous studies have reported that PRS may improve cardiovascular risk discrimination when added to clinical factors. The American Heart Association notes that PRS integration enhances CVD prediction, but clinical applications lack sex-specific investigation (53). Ramírez J et al. found that inclusion of CAD PRS in the clinical model significantly improved the MACE risk stratification (20), while integration of metabolomic scores, PRSs, and 11 clinical biomarkers into the SCORE2 model further enhanced 10-year first-onset CVD discrimination and stratification (54). Similarly, our stepwise integration of lifestyle, metabolic, clinical comorbidity, and PRS into social-demographic scores gradually increased C-statistic from 0.662 to 0.730 in the overall population. However, addition of the polygenic risk score provided only a modest further improvement in males but no significant improvement in females. Although NRI and IDI indicated incremental predictive information, calibration was similar before and after incorporation of polygenic risk, Brier scores decreased only minimally, and DCA showed limited additional net benefit. Among the individual PRSs, CAD PRS produced the largest incremental improvement when entered first in the sequential analysis. However, given the overlap in genetic information captured by CAD PRS and CVD PRS, their relative predictive contributions require further validation in future studies. These findings indicate that the information captured by polygenic risk may differ between females and males. These results suggest that polygenic risk may provide incremental predictive value beyond conventional risk factors, particularly in males. However, whether these findings improve clinically meaningful risk stratification or decision-making requires further evaluation.

Notably, the PAR analyses provided a complementary population-level perspective. Social-demographic characteristics accounted for the largest estimated PAR in both sexes, despite clinical comorbidities conferring the highest relative risk. This discrepancy likely reflects the lower comorbidity prevalence in the relatively healthier and older UK Biobank cohort (55). Cardiomyopathy and OSA were uncommon, yielding less precise sex-specific estimates; however, their near-zero PARs indicated minimal contributions to the population-level MACE burden. Aging, an unavoidable major CVD risk factor (56), aligns with our finding that individuals > 60 years were the largest MACE contributors. While prior global cohorts identified cardiometabolic diseases as top modifiable CVD PAR contributors (57), our study is the first to provide comprehensive, sex-specific PAR data for five risk domains.

Sex differences in PAR were observed: PAR of the polygenic risk was higher in males (15.9%) than females (14.0%). This difference may be associated with sex-specific genetic architecture and warrants further investigation (49). Lifestyle risk accounted for a higher estimated PAR in females (PAR: 15.6%) than in males (13.6%), consistent with prior work (6, 58). Despite known sex differences in metabolism (59, 60), the interaction for the metabolic risk score did not meet the significance threshold (*P* for interaction = 0.065), possibly due to inadequate capture of long-term risk via single baseline measurements. For effective and precise MACE prevention, priorities include promoting population-wide healthy lifestyles, addressing socio-structural inequalities through policy interventions, and stratifying modifiable risk factor management based on risk severity, feasibility, and benefit magnitude across sex strata.

These findings highlight the potential relevance of considering sex when interpreting multidomain cardiovascular risk profiles. For males, polygenic risk scores, particularly CAD PRS, may modestly improve risk stratification beyond clinical factors, suggesting that the role of polygenic risk in sex-aware risk assessment warrants further evaluation. For females, social-demographic disparities, unhealthy lifestyles, and clinical comorbidities drive MACE risk, emphasizing policy-level efforts to address socioeconomic inequalities and targeted lifestyle modification programs. Future research incorporating rare genetic variants and longitudinal metabolic measurements will refine risk profiling. Additionally, investigating younger populations could unlock the potential of polygenic risk scores for early-life risk stratification, enabling proactive prevention. Mechanistic studies into sex-specific genetic-clinical risk interactions in males may identify novel therapeutic targets. Future studies should evaluate whether incorporating sex-specific genetic and clinical information improves risk assessment in external cohorts. This sex-stratified approach to model development warrants prioritization as a key direction for future investigative efforts in MACE risk stratification.

Strengths of this study include its large, prospective UK Biobank cohort (*n* = 397,806) with long-term follow-up (median 15.6 years), comprehensive assessment of five interconnected risk domains, and rigorous statistical validation via multiple sensitivity analyses. The weighted risk score approach and PAR analysis provide actionable insights into relative risk contributions across sexes. Several limitations should also be acknowledged. First, UK Biobank predominantly includes participants of European ancestry and is subject to healthy volunteer bias, limiting the generalizability of the findings and the transportability of PAR estimates. In addition, the standard PRSs used in this study were developed and validated predominantly in European-ancestry populations, and their distributions, effect estimates, and predictive performance may not be directly transferable to non-European ancestry groups. Although the findings were broadly consistent after excluding non-White participants, the PRS-related conclusions require further validation in ancestrally diverse cohorts. Second, lifestyle and metabolic factors were assessed at baseline and may have changed during follow-up. Third, the primary analysis was based on complete cases, and selection bias related to missing data cannot be excluded, although multiple imputation analyses yielded broadly consistent findings. Fourth, continuous variables were categorized using data-derived cutoffs to facilitate construction of interpretable multidomain risk scores. Although this approach enabled integration of heterogeneous risk factors into a unified framework, categorization may reduce information compared with continuous modeling and limit transportability. Future studies using continuous approaches may further refine individualized risk prediction. Finally, the weighted scores were constructed and evaluated within the same cohort. Although five-fold cross-validation and bootstrap optimism correction were performed, variable selection and weight estimation were not repeated independently within each validation iteration; therefore, residual optimism cannot be excluded. External validation in independent cohorts is needed to further assess the reproducibility, transportability, and clinical utility of these risk scores.

## Conclusions

Multidomain cardiovascular risk profiles showed heterogeneous associations, joint patterns, and population-level burden across sexes. Conventional risk domains showed stronger associations with MACE in females, whereas polygenic risk was more strongly associated with MACE and provided modest incremental discrimination in males. The clinical utility of incorporating polygenic risk requires further evaluation in external cohorts.

## Data Availability

Publicly available datasets were analyzed in this study. This data can be found here: The UK Biobank resource is accessible to researchers upon application via https://www.ukbiobank.ac.uk/.
