# Supplementary material for: Sex differences in the associations of multidomain risk factor profiles with incident major adverse cardiovascular events
Source: Front Cardiovasc Med. 2026 Sep 3;13:1911138. doi: 10.3389/fcvm.2026.1911138 (PMC13582396; doi:10.3389/fcvm.2026.1911138)
Supplement: Supplementary file 1 [file Datasheet1.docx]

**Supplemental Material**

**Appendix S1. Candidate risk factor profiles associated with major adverse cardiovascular events.**

The study assessed comprehensive established and potential risk factors associated with major adverse cardiovascular events (MACE) based on previous investigations, encompassing social-demographic characteristics, lifestyle behaviours, metabolic indicators, clinical comorbidities, and polygenic risk. Included older (age ≥ 60), male sex, ethnicity, higher Townsend deprivation index (TDI), less education, current smoker, excessive alcohol intake, longer sleep duration, sedentary, overweight/obesity, higher low-density lipoprotein (LDL), higher triglycerides (TG), higher glycated hemoglobin (HbA1c), lower insulin-like growth factor 1 (IGF-1), diabetes mellitus (DM), hypertension, atrial fibrillation (AF), valvular heart disease (VHD), cardiomyopathy, venous thromboembolism (VTE), hyperthyroidism, dyslipidemia, chronic obstructive pulmonary disease (COPD), chronic kidney disease (CKD), obstructive sleep apnea (OSA), and high polygenic risk scores for AF (AF PRS), cardiovascular disease (CVD PRS), coronary artery disease (CAD PRS), and stroke (Stroke PRS).^1-8^

Significant risk factor profiles determined by least absolute shrinkage and selection operator (LASSO) Cox regression were categorized into 5 aspects:

| **Risk domain** | **Included risk factor profiles** | **Definition/cutoff** | **Weight derivation** |
| --- | --- | --- | --- |
| **Social-demographic risk** | Older (age ≥ 60), higher TDI, and less education | Definitions and category thresholds are provided in Table S4 | β coefficients from multivariable Cox regression models for incident MACE (corresponding HRs presented in Table S6) |
| **Lifestyle risk** | Current smoker, longer sleep duration, and sedentary |  |  |
| **Metabolic risk** | Overweight/obesity, higher TG, higher HbA1c, and lower IGF-1 |  |  |
| **Clinical comorbidity risk** | DM, hypertension, AF, VHD, cardiomyopathy, VTE, COPD, CKD, and OSA |  |  |
| **Polygenic risk** | High AF PRS, high CVD PRS, high CAD PRS and high Stroke PRS | Highest quintile of each PRS |  |

Weighted risk scores were constructed by summing individual risk factors weighted according to β coefficients estimated from multivariable Cox regression models in the overall cohort. Hazard ratios presented in Table S6 correspond to the exponentiated regression coefficients and are provided to illustrate the magnitude of association between individual risk factors and incident MACE. Continuous variables were categorized using optimal cutoffs determined by the maximum Youden index (Table S4).

**Figure S1****. Flowchart of the study population.**

The numbers and proportions of missing data on clinical risk factor profiles are detailed in Table S2.

**Figure S2. Selection of risk factor profiles associated with MACE using the LASSO Cox regression.**

The optimal penalty parameter (λ) was determined via 10-fold cross-validation, with the λ-se value selected to obtain a model with maximized accuracy while using the fewest variables. The blue line indicated the minimal λ and the red line the indicted λ-se. Variables with non-zero coefficients at threshold of λ-se were retained as significant risk factor profiles, including: older (age ≥ 60), male sex, higher TDI, less education, current smoker, longer sleep duration, sedentary, overweight/obesity, higher TG, higher HbA1c, lower IGF-1, DM, hypertension, AF, VHD, cardiomyopathy, VTE, COPD, CKD, OSA, high AF PRS, high CVD PRS, high CAD PRS, and high Stroke PRS. For abbreviations, see Table S1.

**Figure S3. Pairwise correlations among the four polygenic risk scores.**

Pearson correlation coefficients were calculated using the continuous AF, CVD, CAD, and stroke polygenic risk scores. Values within the lower-triangular matrix represent Pearson correlation coefficients.

**Figure S4. Sex-specific associations of continuous risk factors with incident major adverse cardiovascular events modeled using restricted cubic splines.**

Restricted cubic spline models with four knots were used to examine the associations of (A) age, (B) TDI, (C) sleep duration, (D) sedentary time, (E) BMI, (F) LDL, (G) triglycerides, (H) HbA1c, (I) IGF-1, (J) AF PRS, (K) CVD PRS, (L) CAD PRS, and (M) stroke PRS with incident MACE across sex strata.

**Figure S5. Kaplan–Meier cumulative incidence curves for MACE according to the five categorized risk scores in the overall and sex-stratified populations.**

Kaplan–Meier cumulative incidence curves for MACE according to categorized social-demographic (A), lifestyle (B), metabolic (C), clinical comorbidity (D), and polygenic risk (E) scores, with high-risk categories defined by optimal cutoffs in Table S4, in the overall and sex-stratified populations.

**Figure S6. Kaplan–Meier cumulative incidence curves for MACE according to count of the five categorized risk scores in the overall and sex-stratified populations.**

The numbers indicated the count of high-risk categories (range: 0–5) across the five risk scores.

**Figure S7. Interaction Effect among the Five Categorized Risk Scores on MACE Risk in the Overall and Sex-Stratified Populations.**

Interaction effect among the five categorized risk scores on MACE risk in all participants (A), females (B) and males (C). Models were mutually adjusted for individual risk scores and pairwise interactions, with sex additionally adjusted in all participants.

**Figure S8. Incremental predictive value of stepwise combinations of the risk scores for MACE in the overall and sex-stratified populations validated by 5-fold cross-validation.**

ΔC indicated the changes in Harrell’s C-statistic relative to the preceding model. Model 1: social-demographic risk score; Model 2: Model 1 + lifestyle risk score; Model 3: Model 2 + metabolic risk score; Model 4: Model 3 + clinical comorbidity risk score; Model 5: Model 4 + polygenic risk score. *P* values were corrected by Bonferroni (n = 4).

**Figure S9. Decision curves of stepwise combinations of the risk scores for MACE in the overall and sex-stratified populations.**

This figure shows the net benefit of stepwise combined risk scores in all participants (A), females (B), and males (C). The x-axis represents threshold probability and y-axis represents standardized net benefit. Model 1: social-demographic risk score; Model 2: Model 1 + lifestyle risk score; Model 3: Model 2 + metabolic risk score; Model 4: Model 3 + clinical comorbidity risk score; Model 5: Model 4 + polygenic risk score.

**Figure S10. Calibration plots for predicted 10-year MACE risk in the overall and sex-stratified populations.**

Calibration plots compare Model 4 and Model 5 in the overall population (A), females (B), and males (C). Model 4 included social-demographic, lifestyle, metabolic, and clinical comorbidity risk scores, whereas Model 5 additionally included the polygenic risk score. The diagonal line represents perfect agreement between predicted and observed risks. The 10-year Brier scores for Model 4 and Model 5 were 0.04405 and 0.04389 in the overall population, 0.02945 and 0.02940 in females, and 0.06203 and 0.06173 in males, respectively (all *P* < 0.001). Lower Brier scores indicate better overall prediction accuracy.

**Table S1. Variable definitions in UK Biobank.**

| **MACE** | **Field ID** | **Field name(s)** | **Instance(s)** | **Value type** | **Notes** |
| --- | --- | --- | --- | --- | --- |
| Myocardial infarction | 131298,131299,  131300,131301 | Acute myocardial infarction,  subsequent myocardial infarction | - | Date, categorical | I21, I22 (Date and source of first reported) |
| Heart failure | 131354 | Heart failure | - | Date, categorical | I50 (Date and source of first reported) |
| Stroke | 131366,  131368 | Cerebral infarction,  stroke, not specified as hemorrhage or infarction | - | Date, categorical | I63, I64 (Date and source of first reported) |
| Cardiovascular mortality | 40000, 40001, 40002 | Date of death,  underlying (primary) cause of death: ICD10,  contributory (secondary) causes of death: ICD10 | 0,1 | Date, categorical | Date and cause of death |
| **Social-demographic characteristics** | **Field ID** | **Field name(s)** | **Instance(s)** | **Value type** | **Notes** |
| Age | 21022 | Age at recruitment |  | Integer in years |  |
| Sex | 31 | Sex | 0 | 1) Male; 2) Female |  |
| Townsend deprivation index (TDI) | 22189 | Townsend deprivation index at recruitment | - | Continuous |  |
| Ethnicity^*^ | 21000 | Ethnic background | 0 | 1) White; 2) Others | “Mixed”, “Asian”, “Black”, “Chinese”, and “Other ethnic group” were combined into category “Others” given low frequency. |
| Education status^*^ | 6138 | Education qualifications | 0 | 1) College or university degree; 2) Others |  |
| **Lifestyle behaviours** | **Field ID** | **Field name(s)** | **Instance(s)** | **Value type** | **Notes** |
| Smoking status^*^ | 20116,  1239 | Smoking status,  current tobacco smoking | 0 | 1) Current; 2) Non-current | “Previous” and “Never” combined into category “non-current”. |
| Alcohol consumption^*^ | 1558,  1568,  1578,  1588,  1598,  1608,  5364,  4407,  4418,  4429,  4440,  4451,  4462 | Alcohol intake frequency,  average weekly red wine intake,  average weekly champagne plus white wine intake,  average weekly beer plus cider intake,  average weekly spirits intake,  average weekly fortified wine intake,  average weekly intake of other alcoholic drinks,  average monthly red wine intake,  average monthly champagne plus white wine intake,  average monthly beer plus cider intake,  average monthly spirits intake,  average monthly fortified wine intake,  average monthly intake of other alcoholic drinks | 0 | Continuous in standard drinks /week |  |
| Sedentary time^*^ | 1090,  1080,  1070 | Time spent driving,  time spent using computer,  time spent watching television (TV) | 0 | Integer in hours/day |  |
| Sleep duration^*^ | 1160 | Sleep duration | 0 | Integer in hours/day |  |
| **Metabolic indicators** | **Field ID** | **Field name(s)** | **Instance(s)** | **Value type** | **Notes** |
| Body mass index  (BMI)^*^ | 21001 | Body mass index  (BMI) | 0 | Continuous in kg/m^2^ |  |
| Low-density lipoprotein (LDL) | 30780 | LDL direct | 0 | Continuous in mmol/L |  |
| Triglycerides | 30870 | Triglycerides | 0 | Continuous in mmol/L |  |
| Glycated hemoglobin (HbA1c) | 30750 | Glycated hemoglobin (HbA1c) | 0 | Continuous in mmol/mol |  |
| Insulin-like growth factor 1 (IGF-1) | 30770 | IGF-1 | 0 | Continuous in nmol/L |  |
| **Clinical comorbidities** | **Field ID** | **Field name(s)** | **Instance(s)** | **Value type** | **Notes** |
| Hypertension | 131286,  131288,  131290,  131292,  131294 | Essential (primary) hypertension,  hypertensive heart disease,  hypertensive renal disease,  hypertensive heart and renal disease,  secondary hypertension | - | Date | I10, I11, I12, I13, I15 (Date first reported) |
|  | 6177,  6153 | Medication for cholesterol, blood pressure or diabetes;  medication for cholesterol, blood pressure, diabetes, or take exogenous hormones | 0 | Categorical (multiple) | Blood pressure medication |
| Diabetes | 130706,  130708,  130710,  130712,  130714 | Insulin-dependent diabetes mellitus,  non-insulin-dependent diabetes mellitus,  malnutrition-related diabetes mellitus,  other specified diabetes mellitus,  unspecified diabetes mellitus | - | Date | E10, E11, E12, E13, E14 (Date first reported) |
|  | 6177,  6153 | Medication for cholesterol, blood pressure or diabetes;  medication for cholesterol, blood pressure, diabetes, or take exogenous hormones | 0 | Categorical (multiple) | Insulin |
| Valvular heart disease (VHD) | 131276,  131278,  131280,  131282,  131322,  131324,  131326,  131328,  131332 | Rheumatic mitral valve diseases,  rheumatic aortic valve diseases,  rheumatic tricuspid valve diseases,  multiple valve diseases,  nonrheumatic mitral valve disorders,  nonrheumatic aortic valve disorders,  nonrheumatic tricuspid valve disorders,  pulmonary valve disorders,  endocarditis and heart valve disorders in diseases classified elsewhere | - | Date | I05, I06, I07, I08, I34, I35, I36, I37, I39 (Date first reported) |
| Atrial fibrillation (AF) | 131350 | Atrial fibrillation and flutter | - | Date | I48 (Date first reported) |
| Cardiomyopathy | 131338,131340 | Cardiomyopathy,  cardiomyopathy in diseases classified elsewhere | - | Date | I42, I43 (Date first reported) |
| Venous thromboembolism (VTE) | 131308, 131396, 131398, 131400 | Pulmonary embolism,  phlebitis and thrombophlebitis,  portal vein thrombosis,  other venous embolism and thrombosis | - | Date | I26, I80, I81, I82 (Date first reported) |
| Chronic obstructive pulmonary disease (COPD) | 131486, 131488, 131490, 131492 | Simple and mucopurulent chronic bronchitis,  unspecified chronic bronchitis,  emphysema,  other chronic obstructive pulmonary disease | - | Date | J41, J42, J43, J44 (Date first reported) |
| Chronic kidney disease (CKD) | 132032 | Chronic renal failure | - | Date | N18 (Date first reported) |
| Dyslipidemia | 130814 | disorders of lipoprotein metabolism and other lipidaemias | - | Date | E78 (Date first reported) |
| Obstructive sleep apnea (OSA) | 41270,  41280 | Diagnoses - ICD10,  date of first in-patient diagnosis - ICD10 | - | Date | G47.3 Sleep apnea |
| Hyperthyroidism | 130700 | Thyrotoxicosis (hyperthyroidism) | - | Date | E05 (Date first reported) |
| **Polygenic risk scores** | **Field ID** | **Field name(s)** | **Instance(s)** | **Value type** | **Notes** |
| Polygenic risk score for atrial fibrillation (AF PRS) | 26212 | Standard PRS for atrial fibrillation | - | Continuous | relative risk |
| Polygenic risk score for cardiovascular disease (CVD PRS) | 26223 | Standard PRS for cardiovascular disease (CVD) | - | Continuous | relative risk |
| Polygenic risk score for coronary artery disease (CAD PRS) | 26227 | Standard PRS for coronary artery disease (CAD) | - | Continuous | relative risk |
| Polygenic risk score for stroke (Stroke PRS) | 26248 | Standard PRS for ischaemic stroke | - | Continuous | relative risk |
| ^*^Responses “Do not know” and “Prefer not to answer” were considered non-informative (i.e., missing).  Prevalent diseases were defined as those occurring prior to baseline. | | | | | |

**Table S2. Numbers and proportions of missing data on clinical risk factor profiles.**

| **Variable** | **Overall, n (%)** | **Females, n (%)** | **Males, n (%)** | ***P* value** |
| --- | --- | --- | --- | --- |
| **TDI** | 571 (0.12%) | 306 (0.12%) | 265 (0.13%) | 0.414 |
| **Ethnicity** | 2,185 (0.47%) | 1,021 (0.40%) | 1,164 (0.56%) | < 0.001 |
| **BMI** | 1,752 (0.38%) | 888 (0.34%) | 864 (0.41%) | < 0.001 |
| **Education** | 5,881 (1.26%) | 3,230 (1.25%) | 2,651 (1.27%) | 0.569 |
| **Current smoker** | 2,363 (0.51%) | 1,279 (0.50%) | 1,084 (0.52%) | 0.254 |
| **Alcohol consumption** | 1,020 (0.22%) | 533 (0.21%) | 487 (0.23%) | 0.051 |
| **Sleep duration** | 3,428 (0.74%) | 2,189 (0.85%) | 1,239 (0.59%) | < 0.001 |
| **Sedentary time** | 13,950 (2.99%) | 8,424 (3.27%) | 5,523 (2.65%) | < 0.001 |
| **LDL** | 22,677 (4.87%) | 12,635 (4.90%) | 10,042 (4.82%) | 0.185 |
| **Triglycerides** | 22,196 (4.76%) | 12,369 (4.80%) | 9,827 (4.72%) | 0.181 |
| **HbA1c** | 23,252 (4.99%) | 12,870 (5.00%) | 10,382 (4.98%) | 0.852 |
| **IGF-1** | 24,271 (5.21%) | 13,596 (5.28%) | 10,675 (5.12%) | 0.019 |

Baseline missing data for clinical risk factor profiles were imputed under the assumption that data were missing at random, using multiple imputation with the mice package in R. Five imputed datasets were created and pooled results were derived using Rubin’s rules. For abbreviations, see Table S1.

**Table S3. Baseline characteristics of participants included in and excluded from the complete-case analysis.**

|  | **Included**  **N = 397,806** | **Excluded**  **N = 68,150** | ***P* value** | **SMD** |
| --- | --- | --- | --- | --- |
| **Age** | 56.3 (8.1) | 56.8 (8.2) | < 0.001 | 0.060 |
| **Male** | 178,533 (44.9%) | 29,793 (43.7%) | < 0.001 | 0.023 |
| **TDI** | -2.3 [-3.7, 0.3] | -1.8 [-3.5, 1.4] | < 0.001 | 0.183 |
| **Ethnicity (White)** | 379,799 (95.5%) | 58,823 (89.2%) | < 0.001 | 0.238 |
| **BMI** | 27.3 (4.7) | 27.7 (5.0) | < 0.001 | 0.079 |
| **Education** | 263,660 (66.3%) | 43,658 (70.1%) | < 0.001 | 0.082 |
| **Current smoker** | 40,660 (10.2%) | 7,482 (11.4%) | < 0.001 | 0.037 |
| **Alcohol consumption** | 12.8 (15.4) | 11.3 (15.0) | < 0.001 | 0.097 |
| **Sleep duration** | 7.2 (1.1) | 7.1 (1.2) | < 0.001 | 0.025 |
| **Sedentary** | 4.8 (2.4) | 4.9 (2.6) | < 0.001 | 0.035 |
| **LDL** | 3.6 (0.9) | 3.6 (0.9) | < 0.001 | 0.044 |
| **TG** | 1.5 [1.0, 2.1] | 1.5 [1.1, 2.2] | 0.001 | 0.016 |
| **HbA1c** | 35.1 [32.7, 37.7] | 35.6 [33.1, 38.5] | < 0.001 | 0.140 |
| **IGF-1** | 21.3 [17.6, 24.9] | 20.9 [17.1, 24.6] | < 0.001 | 0.075 |
| **Hypertension** | 104,294 (26.2%) | 19,656 (28.8%) | < 0.001 | 0.059 |
| **DM** | 17,859 (4.5%) | 4,121 (6.0%) | < 0.001 | 0.070 |
| **AF** | 4,995 (1.3%) | 948 (1.4%) | 0.004 | 0.012 |
| **VHD** | 2,178 (0.5%) | 413 (0.6%) | 0.061 | 0.008 |
| **Cardiomyopathy** | 396 (0.1%) | 73 (0.1%) | 0.610 | 0.002 |
| **VTE** | 11,013 (2.8%) | 2,162 (3.2%) | < 0.001 | 0.024 |
| **Hyperthyroidism** | 4,271 (1.1%) | 752 (1.1%) | 0.499 | 0.003 |
| **Dyslipidemia** | 52,493 (13.2%) | 10,037 (14.7%) | < 0.001 | 0.044 |
| **COPD** | 6,748 (1.7%) | 1,475 (2.2%) | < 0.001 | 0.034 |
| **CKD** | 4,035 (1.0%) | 869 (1.3%) | < 0.001 | 0.025 |
| **OSA** | 1,700 (0.4%) | 376 (0.6%) | < 0.001 | 0.018 |

Data are presented as mean (SD), median [IQR], or n (%), as appropriate. For participants excluded from the complete-case analysis, percentages were calculated among those with available data for the corresponding variable. SMD, standardized mean difference. For other abbreviations, see Table S1.

**Table S4. Optimal cutoff values of continuous variables determined by ROC.**

| **Variables** | **Direction** | **Cutoff value*** | **AUC** | **Sensitivity** | **Specificity** | **Youden index** |
| --- | --- | --- | --- | --- | --- | --- |
| **Age** | **≥** | 60 | 0.687 | 67.4% | 60.3% | 0.277 |
| **TDI** | **≥** | 0.64 | 0.535 | 27.5% | 77.6% | 0.051 |
| **Alcohol consumption** | **≥** | 23 | 0.504 | 22.1% | 82.1% | 0.042 |
| **Sleep duration** | **≥** | 9 | 0.503 | 10.2% | 92.9% | 0.031 |
| **Sedentary** | **≥** | 5 | 0.564 | 54.6% | 55.5% | 0.101 |
| **LDL** | **≥** | 2.73 | 0.519 | 79.7% | 84.9% | 0.646 |
| **TG** | **≥** | 1.50 | 0.578 | 59.3% | 52.3% | 0.116 |
| **HbA1c** | **≥** | 36.70 | 0.608 | 48.2% | 67.5% | 0.157 |
| **IGF-1** | **≤** | 20.30 | 0.558 | 50.4% | 58.2% | 0.086 |
| **Social-demographic risk score** | **≥** | 2.10 | 0.665 | 67.4% | 60.3% | 0.277 |
| **Lifestyle risk score** | **≥** | 0.33 | 0.577 | 64.6% | 47.7% | 0.123 |
| **Metabolic risk score** | **≥** | 2.23 | 0.624 | 55.0% | 63.4% | 0.184 |
| **Clinical comorbidity risk score** | **≥** | 0.49 | 0.642 | 54.7% | 70.6% | 0.253 |
| **Polygenic risk score** | **≥** | 1.37 | 0.573 | 51.2% | 59.4% | 0.106 |

*The optimal cutoff values of continuous variables were determined via the maximum Youden index using the “cutpointr” package in R. Abbreviations: AUC, area under the curve. For other abbreviations, see Table S1.

**Table S5. Multicollinearity test of risk factors for MACE.**

| **Variable** | **GVIF** | **Adjusted GVIF** |
| --- | --- | --- |
| **Age ≥ 60** | 1.11640 | 1.05660 |
| **Sex (male)** | 1.07995 | 1.03921 |
| **Higher TDI** | 1.04613 | 1.02281 |
| **Less education** | 1.04840 | 1.02392 |
| **Current smoker** | 1.06905 | 1.03395 |
| **Longer sleep** | 1.01392 | 1.00694 |
| **Sedentary** | 1.06434 | 1.03167 |
| **Overweight/obesity** | 1.13610 | 1.06588 |
| **Higher TG** | 1.08683 | 1.04251 |
| **Higher HbA1c** | 1.19020 | 1.09096 |
| **Lower IGF-1** | 1.03898 | 1.01930 |
| **DM** | 1.19751 | 1.09431 |
| **Hypertension** | 1.19728 | 1.09420 |
| **AF** | 1.12796 | 1.06205 |
| **VHD** | 1.07201 | 1.03538 |
| **Cardiomyopathy** | 1.02653 | 1.01318 |
| **VTE** | 1.01231 | 1.00613 |
| **COPD** | 1.03167 | 1.01571 |
| **CKD** | 1.02194 | 1.01091 |
| **OSA** | 1.00805 | 1.00402 |
| **High AF PRS** | 1.04654 | 1.02301 |
| **High CVD PRS** | 1.95068 | 1.39886 |
| **High CAD PRS** | 1.69157 | 1.30060 |
| **High Stroke PRS** | 1.24071 | 1.11387 |

GVIF: generalized variance inflation factors. For other abbreviations, see Table S1.

**Table S6. Univariable and multivariable-adjusted hazard ratios of incident MACE associated with risk scores and risk factor profiles in all participants.**

| **Variable** | **Univarable** | | **Multivariable** | |
| --- | --- | --- | --- | --- |
|  | **HR (95% CI)** | ***P* value** | **HR (95% CI)** | ***P* value** |
| **Social-demographic risk score**^*^ | 1.72 (1.71-1.74) | **< 0.001** | 1.54 (1.52-1.56) | **< 0.001** |
| Age ≥ 60 | 3.08 (3.01-3.16) | **< 0.001** | 2.53 (2.46-2.59) | **< 0.001** |
| Higher TDI | 1.31 (1.27-1.34) | **< 0.001** | 1.21 (1.18-1.24) | **< 0.001** |
| Less education | 1.59 (1.55-1.63) | **< 0.001** | 1.23 (1.20-1.26) | **< 0.001** |
| **Lifestyle risk score**^*^ | 1.38 (1.36-1.39) | **< 0.001** | 1.35 (1.34-1.37) | **< 0.001** |
| Current smoker | 1.80 (1.75-1.86) | **< 0.001** | 1.79 (1.74-1.85) | **< 0.001** |
| Longer sleep | 1.49 (1.44-1.55) | **< 0.001** | 1.16 (1.11-1.20) | **< 0.001** |
| Sedentary | 1.49 (1.45-1.52) | **< 0.001** | 1.09 (1.07-1.12) | **< 0.001** |
| **Metabolic risk score**^*^ | 1.46 (1.44-1.47) | **< 0.001** | 1.18 (1.17-1.19) | **< 0.001** |
| Overweight/obesity | 1.76 (1.72-1.81) | **< 0.001** | 1.20 (1.16-1.23) | **< 0.001** |
| Higher TG | 1.57 (1.54-1.61) | **< 0.001** | 1.11 (1.09-1.14) | **< 0.001** |
| Higher HbA1c | 1.90 (1.86-1.94) | **< 0.001** | 1.22 (1.19-1.25) | **< 0.001** |
| Lower IGF-1 | 1.40 (1.37-1.43) | **< 0.001** | 1.18 (1.15-1.20) | **< 0.001** |
| **Clinical comorbidity risk score**^*^ | 2.30 (2.28-2.33) | **< 0.001** | 1.83 (1.80-1.85) | **< 0.001** |
| DM | 3.04 (2.94-3.14) | **< 0.001** | 1.49 (1.44-1.55) | **< 0.001** |
| Hypertension | 2.56 (2.51-2.62) | **< 0.001** | 1.54 (1.50-1.58) | **< 0.001** |
| AF | 4.40 (4.17-4.64) | **< 0.001** | 2.07 (1.96-2.19) | **< 0.001** |
| VHD | 4.10 (3.78-4.44) | **< 0.001** | 2.29 (2.11-2.49) | **< 0.001** |
| Cardiomyopathy | 5.66 (4.81-6.66) | **< 0.001** | 2.55 (2.16-3.00) | **< 0.001** |
| VTE | 1.89 (1.80-1.99) | **< 0.001** | 1.44 (1.37-1.51) | **< 0.001** |
| COPD | 3.45 (3.28-3.63) | **< 0.001** | 1.98 (1.88-2.09) | **< 0.001** |
| CKD | 2.82 (2.63-3.02) | **< 0.001** | 1.49 (1.39-1.60) | **< 0.001** |
| OSA | 2.36 (2.11-2.65) | **< 0.001** | 1.32 (1.18-1.48) | **< 0.001** |
| **Polygenic risk score**^*^ | 1.49 (1.47-1.52) | **< 0.001** | 1.40 (1.38-1.43) | **< 0.001** |
| High AF PRS | 1.44 (1.39-1.49) | **< 0.001** | 1.25 (1.21-1.30) | **< 0.001** |
| High CVD PRS | 1.85 (1.78-1.92) | **< 0.001** | 1.34 (1.28-1.40) | **< 0.001** |
| High CAD PRS | 1.76 (1.70-1.83) | **< 0.001** | 1.38 (1.32-1.44) | **< 0.001** |
| High Stroke PRS | 1.66 (1.60-1.72) | **< 0.001** | 1.16 (1.11-1.20) | **< 0.001** |

*HR indicated the risk of incident MACE associated with a per 1-point increase for 5 weighted risk scores. Multivariable Cox model was mutually adjusted for individual risk factors and sex. Statistical significance was defined as a Bonferroni-corrected threshold of *P* < 0.002 (0.05/28). For abbreviations, see Table S1.

**Table S7. Numbers of incident MACE events among participants with individual clinical comorbidities.**

|  | **Clinical comorbidities** | **MACE events / participants with comorbidities** |
| --- | --- | --- |
| **Overall** | **DM** | 3,671 / 17,859 (20.6%) |
|  | **Hypertension** | 14,795 / 104,294 (14.2%) |
|  | **AF** | 1,465 / 4,995 (29.3%) |
|  | **VHD** | 616 / 2,178 (28.3%) |
|  | **Cardiomyopathy** | 146 / 396 (36.9%) |
|  | **VTE** | 1,557 / 11,013 (14.1%) |
|  | **COPD** | 1,548 / 6,748 (22.9%) |
|  | **CKD** | 818 / 4,035 (20.3%) |
|  | **OSA** | 299 / 1,700 (17.6%) |
| **Females** | **DM** | 1,126 / 6,934 (16.2%) |
|  | **Hypertension** | 5,410 / 50,473 (10.7%) |
|  | **AF** | 447 / 1,637 (27.3%) |
|  | **VHD** | 264 / 1,118 (23.6%) |
|  | **Cardiomyopathy** | 35 / 120 (29.2%) |
|  | **VTE** | 758 / 6,770 (11.2%) |
|  | **COPD** | 613 / 3,347 (18.3%) |
|  | **CKD** | 329 / 2,226 (14.8%) |
|  | **OSA** | 61 / 421 (14.5%) |
| **Males** | **DM** | 2,545 / 10,925 (23.3%) |
|  | **Hypertension** | 9,385 / 53,821 (17.4%) |
|  | **AF** | 1,018 / 3,358 (30.3%) |
|  | **VHD** | 352 / 1,060 (33.2%) |
|  | **Cardiomyopathy** | 111 / 276 (40.2%) |
|  | **VTE** | 799 / 4,243 (18.8%) |
|  | **COPD** | 935 / 3,401 (27.5%) |
|  | **CKD** | 489 / 1,809 (27.0%) |
|  | **OSA** | 238 / 1,279 (18.6%) |

Data are presented as the number of incident MACE events / number of participants with the corresponding comorbidity (event rate).

**Table S8. Multivariable-adjusted hazard ratios of incident MACE associated with risk scores and risk factor profiles across sex strata.**

|  | **Females** | **Males** | **Multiplicative interaction** | |
| --- | --- | --- | --- | --- |
|  | **HR (95% CI)** | **HR (95% CI)** | **Modification effect** | ***P* for interaction** |
| **Social-demographic risk score*** | 1.65 (1.62-1.68) | 1.50 (1.48-1.52) | 0.91 (0.89-0.93) | **< 0.001** |
| Age ≥ 60 | 2.92 (2.80-3.04) | 2.32 (2.25-2.39) | 0.80 (0.76-0.84) | **< 0.001** |
| Higher TDI | 1.23 (1.18-1.28) | 1.19 (1.15-1.23) | 0.97 (0.92-1.02) | 0.269 |
| Less education | 1.21 (1.15-1.26) | 1.24 (1.20-1.28) | 1.03 (0.97-1.08) | 0.373 |
| **Lifestyle risk score*** | 1.37 (1.34-1.40) | 1.28 (1.26-1.31) | 0.94 (0.91-0.97) | **< 0.001** |
| Current smoker | 2.02 (1.92-2.13) | 1.68 (1.62-1.74) | 0.84 (0.78-0.89) | **< 0.001** |
| Longer sleep duration | 1.13 (1.06-1.19) | 1.18 (1.13-1.24) | 1.05 (0.98-1.14) | 0.172 |
| Sedentary | 1.09 (1.05-1.13) | 1.09 (1.06-1.12) | 1.00 (0.96-1.05) | 0.975 |
| **Metabolic risk score*** | 1.18 (1.16-1.20) | 1.16 (1.14-1.18) | 0.98 (0.96-1.00) | 0.065 |
| Overweight/obesity | 1.22 (1.17-1.27) | 1.17 (1.12-1.21) | 0.96 (0.91-1.01) | 0.137 |
| Higher TG | 1.17 (1.13-1.21) | 1.06 (1.03-1.09) | 0.90 (0.86-0.95) | **< 0.001** |
| Higher HbA1c | 1.16 (1.12-1.21) | 1.25 (1.21-1.28) | 1.07 (1.02-1.13) | 0.005 |
| Lower IGF-1 | 1.20 (1.16-1.25) | 1.16 (1.12-1.19) | 0.96 (0.92-1.01) | 0.119 |
| **Clinical comorbidity risk score*** | 1.97 (1.92-2.02) | 1.65 (1.62-1.69) | 0.85 (0.82-0.87) | **< 0.001** |
| DM | 1.69 (1.58-1.81) | 1.42 (1.36-1.49) | 0.84 (0.78-0.91) | **< 0.001** |
| Hypertension | 1.70 (1.63-1.77) | 1.45 (1.41-1.50) | 0.86 (0.82-0.90) | **< 0.001** |
| AF | 2.73 (2.47-3.02) | 1.91 (1.79-2.04) | 0.71 (0.63-0.80) | **< 0.001** |
| VHD | 2.65 (2.33-3.01) | 2.04 (1.83-2.28) | 0.78 (0.66-0.92) | 0.003 |
| Cardiomyopathy | 2.85 (2.04-3.98) | 2.57 (2.13-3.11) | 0.91 (0.62-1.34) | 0.644 |
| VTE | 1.51 (1.41-1.63) | 1.36 (1.26-1.46) | 0.90 (0.81-1.00) | 0.044 |
| COPD | 2.13 (1.96-2.31) | 1.89 (1.76-2.02) | 0.90 (0.80-1.00) | 0.041 |
| CKD | 1.38 (1.23-1.54) | 1.55 (1.42-1.70) | 1.13 (0.98-1.31) | 0.087 |
| OSA | 1.65 (1.28-2.13) | 1.28 (1.13-1.46) | 0.78 (0.59-1.03) | 0.083 |
| **Polygenic risk score*** | 1.36 (1.33-1.40) | 1.46 (1.43-1.49) | 1.07 (1.03-1.11) | **< 0.001** |
| High AF PRS | 1.32 (1.24-1.39) | 1.22 (1.16-1.27) | 0.93 (0.86-1.00) | 0.038 |
| High CVD PRS | 1.20 (1.11-1.29) | 1.43 (1.35-1.52) | 1.19 (1.08-1.31) | **< 0.001** |
| High CAD PRS | 1.30 (1.21-1.40) | 1.43 (1.35-1.51) | 1.10 (1.01-1.20) | 0.038 |
| High Stroke PRS | 1.21 (1.13-1.29) | 1.12 (1.07-1.18) | 0.93 (0.86-1.00) | 0.065 |

*HR indicated the risk of incident MACE associated with a per 1-point increase for 5 weighted risk scores. Models were mutually adjusted for individual risk factors. Statistical significance was defined as a Bonferroni-corrected threshold of *P* < 0.002 (0.05/28). For abbreviations, see Table S1.

**Table S9. Multivariable-adjusted hazard ratios of incident MACE associated with risk scores across sex strata excluding incident MACE cases within the first 2 years of follow-up.**

|  | **HR (95% CI)** | ***P* for interaction** |
| --- | --- | --- |
| **Social-demographic risk score** | | |
| Females | 1.66 (1.63-1.69) | **< 0.001** |
| Males | 1.50 (1.48-1.52) |  |
| **Lifestyle risk score** | | |
| Females | 1.36 (1.33-1.39) | **< 0.001** |
| Males | 1.28 (1.25-1.30) |  |
| **Metabolic risk score** | | |
| Females | 1.18 (1.16-1.20) | 0.057 |
| Males | 1.16 (1.14-1.17) |  |
| **Clinical comorbidity risk score** | | |
| Females | 1.95 (1.90-2.00) | **< 0.001** |
| Males | 1.65 (1.62-1.68) |  |
| **Polygenic risk score** | | |
| Females | 1.36 (1.32-1.40) | **0.002** |
| Males | 1.44 (1.41-1.48) |  |

Model was mutually adjusted for individual risk scores. HR indicated the risk of incident MACE associated with a per 1-point increase. Statistical significance was defined using Bonferroni-corrected thresholds of *P* for interaction < 0.01 (0.05/5).

**Table S10. Multivariable-adjusted hazard ratios of incident MACE associated with risk scores across sex strata excluding of incident MACE cases ascertained by self-reporting.**

|  | **HR (95% CI)** | ***P* for interaction** |
| --- | --- | --- |
| **Social-demographic risk score** | | |
| Females | 1.67 (1.63-1.70) | **< 0.001** |
| Males | 1.52 (1.50-1.54) |  |
| **Lifestyle risk score** | | |
| Females | 1.37 (1.34-1.41) | **< 0.001** |
| Males | 1.29 (1.27-1.31) |  |
| **Metabolic risk score** | | |
| Females | 1.19 (1.17-1.21) | 0.098 |
| Males | 1.16 (1.15-1.18) |  |
| **Clinical comorbidity risk score** | | |
| Females | 1.98 (1.93-2.03) | **< 0.001** |
| Males | 1.67 (1.64-1.70) |  |
| **Polygenic risk score** | | |
| Females | 1.36 (1.32-1.40) | **< 0.001** |
| Males | 1.45 (1.42-1.48) |  |

Model was mutually adjusted for individual risk scores. HR indicated the risk of incident MACE associated with a per 1-point increase. Statistical significance was defined using Bonferroni-corrected thresholds of *P* for interaction < 0.01 (0.05/5).

**Table S11. Multivariable-adjusted hazard ratios of incident MACE associated with risk scores across sex strata excluding non-White participants.**

|  | **HR (95% CI)** | ***P* for interaction** |
| --- | --- | --- |
| **Social-demographic risk score** | | |
| Females | 1.65 (1.62-1.68) | **< 0.001** |
| Males | 1.50 (1.48-1.52) |  |
| **Lifestyle risk score** | | |
| Females | 1.37 (1.34-1.41) | **< 0.001** |
| Males | 1.29 (1.27-1.31) |  |
| **Metabolic risk score** | | |
| Females | 1.18 (1.16-1.20) | 0.219 |
| Males | 1.16 (1.15-1.18) |  |
| **Clinical comorbidity risk score** | | |
| Females | 1.96 (1.91-2.01) | **< 0.001** |
| Males | 1.65 (1.61-1.68) |  |
| **Polygenic risk score** | | |
| Females | 1.37 (1.33-1.41) | **< 0.001** |
| Males | 1.46 (1.43-1.50) |  |

Model was mutually adjusted for individual risk scores. HR indicated the risk of incident MACE associated with a per 1-point increase. Statistical significance was defined using Bonferroni-corrected thresholds of *P* for interaction < 0.01 (0.05/5).

**Table S12. Multivariable-adjusted hazard ratios of incident MACE associated with risk scores across sex strata considering non-cardiovascular mortality as a competing risk.**

|  | **HR (95% CI)** | ***P* for interaction** |
| --- | --- | --- |
| **Social-demographic risk score** | | |
| Females | 1.63 (1.60-1.66) | **< 0.001** |
| Males | 1.47 (1.45-1.49) |  |
| **Lifestyle risk score** | | |
| Females | 1.34 (1.31-1.37) | **< 0.001** |
| Males | 1.26 (1.24-1.28) |  |
| **Metabolic risk score** | | |
| Females | 1.18 (1.16-1.20) | 0.083 |
| Males | 1.16 (1.14-1.18) |  |
| **Clinical comorbidity risk score** | | |
| Females | 1.95 (1.89-2.00) | **< 0.001** |
| Males | 1.63 (1.60-1.67) |  |
| **Polygenic risk score** | | |
| Females | 1.37 (1.33-1.41) | **< 0.001** |
| Males | 1.46 (1.43-1.50) |  |

Model was mutually adjusted for individual risk scores. HR indicated the risk of incident MACE associated with a per 1-point increase. Statistical significance was defined using Bonferroni-corrected thresholds of *P* for interaction < 0.01 (0.05/5).

**Table S13. Multivariable-adjusted hazard ratios of incident MACE associated with risk scores across sex strata after multiple imputation.**

|  | **HR (95% CI)** | ***P* for interaction** |
| --- | --- | --- |
| **Social-demographic risk score** | | |
| Females | 1.64 (1.61-1.66) | **< 0.001** |
| Males | 1.50 (1.48-1.52) |  |
| **Lifestyle risk score** | | |
| Females | 1.36 (1.33-1.39) | **< 0.001** |
| Males | 1.29 (1.27-1.31) |  |
| **Metabolic risk score** | | |
| Females | 1.17 (1.16-1.19) | 0.027 |
| Males | 1.15 (1.13-1.16) |  |
| **Clinical comorbidity risk score** | | |
| Females | 1.99 (1.95-2.04) | **< 0.001** |
| Males | 1.67 (1.64-1.69) |  |
| **Polygenic risk score** | | |
| Females | 1.36 (1.33-1.40) | **< 0.001** |
| Males | 1.46 (1.43-1.49) |  |

Model was mutually adjusted for individual risk scores. HR indicated the risk of incident MACE associated with a per 1-point increase. Statistical significance was defined using Bonferroni-corrected thresholds of *P* for interaction < 0.01 (0.05/5).

**Table S14. Multivariable-adjusted associations of risk scores with incident MACE excluding heart failure across sex strata.**

|  | **HR (95% CI)** | ***P* for interaction** |
| --- | --- | --- |
| **Social-demographic risk score** | | |
| Females | 1.58 (1.54-1.61) | **< 0.001** |
| Males | 1.45 (1.43-1.48) |  |
| **Lifestyle risk score** | | |
| Females | 1.42 (1.38-1.45) | **< 0.001** |
| Males | 1.31 (1.29-1.34) |  |
| **Metabolic risk score** | | |
| Females | 1.16 (1.14-1.18) | 0.196 |
| Males | 1.14 (1.13-1.16) |  |
| **Clinical comorbidity risk score** | | |
| Females | 1.71 (1.67-1.75) | **< 0.001** |
| Males | 1.51 (1.49-1.54) |  |
| **Polygenic risk score** | | |
| Females | 1.41 (1.36-1.45) | **< 0.001** |
| Males | 1.51 (1.48-1.55) |  |

MACE excluding heart failure was defined as a composite outcome of myocardial infarction, stroke, and cardiovascular mortality. Model was mutually adjusted for individual risk scores. HR indicated the risk of incident MACE associated with a per 1-point increase. Statistical significance was defined using Bonferroni-corrected thresholds of *P* for interaction < 0.01 (0.05/5).

**Table S15. Multivariable-adjusted associations of risk scores with incident MACE across sex strata with HbA1c excluded from the metabolic risk score.**

|  | **HR (95% CI)** | ***P* for interaction** |
| --- | --- | --- |
| **Social-demographic risk score** | | |
| Females | 1.67 (1.64-1.70) | **< 0.001** |
| Males | 1.52 (1.50-1.54) |  |
| **Lifestyle risk score** | | |
| Females | 1.38 (1.35-1.41) | **< 0.001** |
| Males | 1.30 (1.27-1.32) |  |
| **Metabolic risk score** | | |
| Females | 1.20 (1.18-1.23) | **< 0.001** |
| Males | 1.14 (1.12-1.16) |  |
| **Clinical comorbidity risk score** | | |
| Females | 1.99 (1.94-2.04) | **< 0.001** |
| Males | 1.69 (1.66-1.72) |  |
| **Polygenic risk score** | | |
| Females | 1.36 (1.33-1.40) | **< 0.001** |
| Males | 1.46 (1.43-1.49) |  |

Model was mutually adjusted for individual risk scores. HR indicated the risk of incident MACE associated with a per 1-point increase. Statistical significance was defined using Bonferroni-corrected thresholds of *P* for interaction < 0.01 (0.05/5).

**Table S16. Multivariable-adjusted associations of domain-specific risk scores with individual components of MACE across sex strata.**

|  | | **HR (95% CI)** | ***P* for interaction** |
| --- | --- | --- | --- |
| **HF** | **Social-demographic risk score** | | |
|  | Females | 1.77 (1.72-1.82) | **0.002** |
|  | Males | 1.66 (1.62-1.70) |  |
|  | **Lifestyle risk score** | | |
|  | Females | 1.32 (1.27-1.37) | 0.047 |
|  | Males | 1.26 (1.22-1.29) |  |
|  | **Metabolic risk score** | | |
|  | Females | 1.22 (1.19-1.25) | 0.013 |
|  | Males | 1.16 (1.14-1.19) |  |
|  | **Clinical comorbidity risk score** | | |
|  | Females | 2.38 (2.31-2.46) | **< 0.001** |
|  | Males | 2.06 (2.00-2.11) |  |
|  | **Polygenic risk score** | | |
|  | Females | 1.29 (1.23-1.35) | 0.362 |
|  | Males | 1.33 (1.28-1.38) |  |
| **MI** | **Social-demographic risk score** | | |
|  | Females | 1.49 (1.44-1.54) | **< 0.001** |
|  | Males | 1.30 (1.28-1.33) |  |
|  | **Lifestyle risk score** | | |
|  | Females | 1.49 (1.43-1.55) | **< 0.001** |
|  | Males | 1.28 (1.25-1.32) |  |
|  | **Metabolic risk score** | | |
|  | Females | 1.22 (1.18-1.26) | 0.994 |
|  | Males | 1.22 (1.19-1.25) |  |
|  | **Clinical comorbidity risk score** | | |
|  | Females | 1.71 (1.62-1.79) | **< 0.001** |
|  | Males | 1.32 (1.28-1.37) |  |
|  | **Polygenic risk score** | | |
|  | Females | 1.80 (1.70-1.90) | **0.006** |
|  | Males | 1.98 (1.92-2.05) |  |
| **Stroke** | **Social-demographic risk score** | | |
|  | Females | 1.68 (1.62-1.74) | 0.048 |
|  | Males | 1.60 (1.56-1.65) |  |
|  | **Lifestyle risk score** | | |
|  | Females | 1.30 (1.24-1.35) | 0.525 |
|  | Males | 1.27 (1.23-1.31) |  |
|  | **Metabolic risk score** | | |
|  | Females | 1.15 (1.12-1.19) | **0.008** |
|  | Males | 1.09 (1.06-1.12) |  |
|  | **Clinical comorbidity risk score** | | |
|  | Females | 1.77 (1.68-1.86) | **< 0.001** |
|  | Males | 1.53 (1.47-1.59) |  |
|  | **Polygenic risk score** | | |
|  | Females | 1.40 (1.33-1.48) | 0.515 |
|  | Males | 1.37 (1.30-1.43) |  |
| **Cardiovascular mortality** | **Social-demographic risk score** | | |
|  | Females | 1.88 (1.82-1.95) | 0.015 |
|  | Males | 1.78 (1.73-1.82) |  |
|  | **Lifestyle risk score** | | |
|  | Females | 1.54 (1.49-1.60) | **0.003** |
|  | Males | 1.43 (1.40-1.47) |  |
|  | **Metabolic risk score** | | |
|  | Females | 1.17 (1.14-1.21) | 0.726 |
|  | Males | 1.18 (1.15-1.21) |  |
|  | **Clinical comorbidity risk score** | | |
|  | Females | 2.18 (2.10-2.27) | **< 0.001** |
|  | Males | 2.00 (1.94-2.05) |  |
|  | **Polygenic risk score** | | |
|  | Females | 1.27 (1.20-1.34) | 0.235 |
|  | Males | 1.32 (1.27-1.37) |  |

Models were mutually adjusted for all five domain-specific risk scores. HRs represent the risk associated with each 1-point increase in the corresponding score. *P* values for interaction were derived from interaction terms between sex and each risk score. Statistical significance was defined using a Bonferroni-corrected threshold of *P* for interaction < 0.01 within each outcome.

**Table S17. Net reclassification improvement and integrated discrimination improvement of stepwise combinations of the risk scores for MACE in the overall and sex-stratified populations.**

|  | | **NRI (95% CI)** | ***P* value** | **IDI (95% CI)** | ***P* value** |
| --- | --- | --- | --- | --- | --- |
| **All participants** | **Model 2 vs Model 1** | 0.175 (0.169-0.182) | **< 0.001** | 0.136 (0.128-0.143) | **< 0.001** |
|  | **Model 3 vs Model 2** | 0.225 (0.218-0.233) | **< 0.001** | 0.134 (0.128-0.140) | **< 0.001** |
|  | **Model 4 vs Model 3** | 0.370 (0.362-0.378) | **< 0.001** | 0.615 (0.585-0.644) | **< 0.001** |
|  | **Model 5 vs Model 4** | 0.200 (0.192-0.208) | **< 0.001** | 0.111 (0.101-0.120) | **< 0.001** |
| **Females** | **Model 2 vs Model 1** | 0.235 (0.223-0.247) | **< 0.001** | 0.164 (0.149-0.178) | **< 0.001** |
|  | **Model 3 vs Model 2** | 0.268 (0.256-0.281) | **< 0.001** | 0.174 (0.162-0.187) | **< 0.001** |
|  | **Model 4 vs Model 3** | 0.380 (0.367-0.392) | **< 0.001** | 0.853 (0.789-0.917) | **< 0.001** |
|  | **Model 5 vs Model 4** | 0.200 (0.187-0.213) | **< 0.001** | 0.113 (0.095-0.130) | **< 0.001** |
| **Males** | **Model 2 vs Model 1** | 0.171 (0.162-0.180) | **< 0.001** | 0.080 (0.073-0.086) | **< 0.001** |
|  | **Model 3 vs Model 2** | 0.237 (0.226-0.247) | **< 0.001** | 0.093 (0.087-0.099) | **< 0.001** |
|  | **Model 4 vs Model 3** | 0.336 (0.326-0.346) | **< 0.001** | 0.379 (0.356-0.403) | **< 0.001** |
|  | **Model 5 vs Model 4** | 0.214 (0.204-0.225) | **< 0.001** | 0.115 (0.105-0.125) | **< 0.001** |

Model 1: social-demographic risk score; Model 2: Model 1 + lifestyle risk score; Model 3: Model 2 + metabolic risk score; Model 4: Model 3 + clinical comorbidity risk score; Model 5: Model 4 + polygenic risk score. Statistical significance was defined using Bonferroni-corrected thresholds of *P* < 0.013 (0.05/4). NRI: net reclassification improvement; IDI: integrated discrimination improvement.

**Table S18. Incremental predictive value of stepwise combined polygenic risk scores for MACE risk in the overall and sex-stratified populations.**

|  | **All participants** | | **Females** | | **Males** | |
| --- | --- | --- | --- | --- | --- | --- |
|  | C-**statistic (95% CI)** | ***P* value** | C-**statistic (95% CI)** | ***P* value** | C-**statistic (95% CI)** | ***P* value** |
| **Clinical model** | 0.723 (0.720-0.725) |  | 0.744 (0.740-0.748) |  | 0.698 (0.694-0.701) |  |
| **Clinical model + CAD PRS** | 0.729 (0.726-0.731) | **0.002** | 0.747 (0.743-0.751) | 0.235 | 0.707 (0.704-0.711) | **< 0.001** |
| **Clinical model + CAD PRS + AF PRS** | 0.730 (0.727-0.732) | 0.554 | 0.749 (0.745-0.753) | 0.626 | 0.709 (0.705-0.712) | 0.649 |
| **Clinical model + CAD PRS + AF PRS + Stroke PRS** | 0.730 (0.728-0.733) | 0.752 | 0.750 (0.746-0.754) | 0.748 | 0.709 (0.706-0.713) | 0.805 |
| **Clinical model + CAD PRS + AF PRS + Stroke PRS + CVD PRS** | 0.731 (0.728-0.733) | 0.790 | 0.750 (0.746-0.754) | 0.947 | 0.710 (0.707-0.714) | 0.721 |

Reference: the preceding model. Clinical model included social-demographic risk score, lifestyle risk score, metabolic risk score, and clinical comorbidity risk score. Statistical significance was defined using Bonferroni-corrected thresholds of *P* < 0.013 (0.05/4).

**Table S19. Population-attributable risk of incident MACE for risk scores across overall and sex-stratified populations.**

|  | **All participants** | | **Females** | | **Males** | |
| --- | --- | --- | --- | --- | --- | --- |
| **Order** | **Risk scores** | **PAR (%)** | **Risk scores** | **PAR (%)** | **Risk scores** | **PAR (%)** |
| **1** | Social-demographic risk | 38.5 (36.8-40.2) | Social-demographic risk | 44.2 (40.8-47.6) | Social-demographic risk | 35.8 (33.9-37.7) |
| **2** | Clinical comorbidity risk | 23.7 (22.5-25.0) | Clinical comorbidity risk | 27.0 (24.6-29.4) | Clinical comorbidity risk | 19.2 (18.0-20.3) |
| **3** | Lifestyle risk | 18.7 (18.3-19.2) | Lifestyle risk | 15.6 (15.0-16.2) | Polygenic risk | 15.9 (15.3-16.6) |
| **4** | Polygenic risk | 14.5 (14.0-15.0) | Metabolic risk | 14.5 (13.6-15.4) | Lifestyle risk | 13.6 (13.1-14.0) |
| **5** | Metabolic risk | 13.7 (13.2-14.2) | Polygenic risk | 14.0 (13.2-14.7) | Metabolic risk | 12.5 (11.9-13.1) |

Models were mutually adjusted for individual risk factor scores, with sex additionally adjusted in all participants.

**Table S20. Population-attributable risk of incident MACE for risk factor profiles in all participants.**

| **Order** | **Variable** | **PAR (%)** | **95% CI** |
| --- | --- | --- | --- |
| **1** | **Age ≥ 60** | 38.9 | 37.2 - 40.6 |
| **2** | **Less education** | 13.3 | 13.0 -13.5 |
| **3** | **Hypertension** | 12.7 | 12.0 - 13.4 |
| **4** | **Overweight/obesity** | 11.6 | 11.3 - 11.8 |
| **5** | **Current smoker** | 7.5 | 6.4 - 8.6 |
| **6** | **Lower IGF-1** | 6.9 | 6.7 - 7.1 |
| **7** | **Higher HbA1c** | 6.8 | 6.6 - 7.1 |
| **8** | **Higher TG** | 5.3 | 5.1 - 5.4 |
| **9** | **High CAD PRS** | 4.7 | 4.4 - 5.1 |
| **10** | **High CVD PRS** | 4.6 | 4.3 - 4.9 |
| **11** | **Higher TDI** | 4.5 | 4.3 - 4.8 |
| **12** | **Sedentary** | 4.1 | 4.0 - 4.2 |
| **13** | **High AF PRS** | 3.2 | 3.1 - 3.4 |
| **14** | **High Stroke PRS** | 2.3 | 2.1 - 2.4 |
| **15** | **DM** | 2.2 | 1.6 - 2.7 |
| **16** | **COPD** | 1.6 | 0.4 - 2.9 |
| **17** | **AF** | 1.3 | 0.0 - 2.7 |
| **18** | **VTE** | 1.2 | 0.7 - 1.7 |
| **19** | **Longer sleep** | 1.1 | 1.0 - 1.3 |
| **20** | **VHD** | 0.7 | -1.1 - 2.5 |
| **21** | **CKD** | 0.5 | 0.0 - 1.0 |
| **22** | **Cardiomyopathy** | 0.2 | -1.9 - 2.2 |
| **23** | **OSA** | 0.1 | -0.2 - 0.5 |

Models were mutually adjusted for individual risk factor profiles and sex. For abbreviations, see Table S1.

**Table S21. Population-attributable risk of incident MACE for risk factor profiles in males.**

| **Order** | **Variable** | **PAR (%)** | **95% CI** |
| --- | --- | --- | --- |
| **1** | **Age ≥ 60** | 35.4 | 33.5 - 37.3 |
| **2** | **Less education** | 13.7 | 13.4 - 14.1 |
| **3** | **Hypertension** | 10.9 | 10.2 - 11.6 |
| **4** | **Overweight/obesity** | 10.0 | 9.7 - 10.3 |
| **5** | **Higher HbA1c** | 7.7 | 7.3 - 8.1 |
| **6** | **Current smoker** | 6.5 | 5.4 - 7.7 |
| **7** | **Lower IGF-1** | 6.2 | 6.0 - 6.4 |
| **8** | **High CVD PRS** | 5.5 | 5.0 - 6.1 |
| **9** | **High CAD PRS** | 5.0 | 4.5 - 5.4 |
| **10** | **Higher TDI** | 4.1 | 3.9 - 4.4 |
| **11** | **Sedentary** | 4.0 | 3.9 - 4.1 |
| **12** | **High AF PRS** | 2.8 | 2.6 - 3.0 |
| **13** | **Higher TG** | 2.8 | 2.7 - 2.9 |
| **14** | **High Stroke PRS** | 1.9 | 1.8 - 2.0 |
| **15** | **DM** | 1.8 | 1.3 - 2.4 |
| **16** | **COPD** | 1.5 | 0.1 - 2.9 |
| **17** | **Longer sleep** | 1.3 | 1.1 - 1.6 |
| **18** | **AF** | 1.1 | -0.1 - 2.4 |
| **19** | **VTE** | 1.0 | 0.4 - 1.6 |
| **20** | **VHD** | 0.6 | -1.1 - 2.3 |
| **21** | **CKD** | 0.6 | -0.2 - 1.4 |
| **22** | **Cardiomyopathy** | 0.2 | -2.2 - 2.5 |
| **23** | **OSA** | 0.1 | -0.2 - 0.4 |

Models were mutually adjusted for individual risk factor profiles. For abbreviations, see Table S1.

**Table S22. Population-attributable risk of incident MACE for risk factor profiles in females.**

| **Order** | **Variable** | **PAR (%)** | **95% CI** |
| --- | --- | --- | --- |
| **1** | **Age ≥ 60** | 44.5 | 41.0 - 48.0 |
| **2** | **Hypertension** | 15.8 | 14.3 - 17.3 |
| **3** | **Overweight/obesity** | 12.7 | 12.2 - 13.1 |
| **4** | **Less education** | 12.3 | 11.9 - 12.7 |
| **5** | **Current smoker** | 9.5 | 6.8 - 12.1 |
| **6** | **Lower IGF-1** | 7.9 | 7.5 - 8.2 |
| **7** | **Higher TG** | 7.7 | 7.4 - 8.0 |
| **8** | **Higher HbA1c** | 5.2 | 4.9 - 5.5 |
| **9** | **Higher TDI** | 4.9 | 4.5 - 5.4 |
| **10** | **High CAD PRS** | 4.2 | 3.8 - 4.7 |
| **11** | **High AF PRS** | 4.2 | 3.8 - 4.6 |
| **12** | **Sedentary** | 4.0 | 3.8 - 4.2 |
| **13** | **High CVD PRS** | 3.0 | 2.7 - 3.4 |
| **14** | **DM** | 3.0 | 1.5 - 4.5 |
| **15** | **High Stroke PRS** | 2.7 | 2.4 - 2.9 |
| **16** | **AF** | 2.1 | -3.0 - 7.2 |
| **17** | **COPD** | 1.9 | -0.6 - 4.4 |
| **18** | **VTE** | 1.4 | 0.5 - 2.3 |
| **19** | **Longer sleep** | 0.9 | 0.7 - 1.1 |
| **20** | **VHD** | 0.9 | -3.2 - 5.0 |
| **21** | **CKD** | 0.4 | -0.2 - 0.9 |
| **22** | **OSA** | 0.3 | -1.5 - 2.0 |
| **23** | **Cardiomyopathy** | 0.2 | -5.5 - 5.9 |

Models were mutually adjusted for individual risk factor profiles. For abbreviations, see Table S1.

**Table S23. Population-attributable risk of incident MACE for risk scores across overall and sex-stratified populations with HbA1c excluded from the metabolic risk score.**

|  | **All participants** | | **Females** | | **Males** | |
| --- | --- | --- | --- | --- | --- | --- |
| **Order** | **Risk scores** | **PAR (%)** | **Risk scores** | **PAR (%)** | **Risk scores** | **PAR (%)** |
| **1** | Social-demographic risk | 39.6 (37.9–41.3) | Social-demographic risk | 45.4 (41.9–48.9) | Social-demographic risk | 36.7 (34.8–38.7) |
| **2** | Clinical comorbidity risk | 25.1 (23.8–26.4) | Clinical comorbidity risk | 28.1 (25.6–30.7) | Clinical comorbidity risk | 20.8 (19.5–22.1) |
| **3** | Metabolic risk | 18.6 (18.1–19.1) | Metabolic risk | 18.3 (17.6–19.1) | Polygenic risk | 16.1 (15.4–16.8) |
| **4** | Polygenic risk | 14.6 (14.1–15.1) | Polygenic risk | 14.0 (13.2–14.7) | Lifestyle risk | 9.5 (8.5–10.4) |
| **5** | Lifestyle risk | 10.2 (9.3–11.1) | Lifestyle risk | 10.3 (8.8–11.8) | Metabolic risk | 13.2 (12.8–13.6) |

Models were mutually adjusted for individual risk factor scores, with sex additionally adjusted in all participants.

**Reference**

1. Global, Regional, and National Burden of Cardiovascular Diseases and Risk Factors in 204 Countries and Territories, 1990-2023. *J Am Coll Cardiol*. 2025;86(22):2167-2243.
2. Ajufo E, Kany S, Rämö JT, et al. Accelerometer-Measured Sedentary Behavior and Risk of Future Cardiovascular Disease. J Am Coll Cardiol. 2024;85(5):473-486.
3. Huang ZG, Gao JW, Chen ZT, et al. Comprehensive Multiple Risk Factor Control in Type 2 Diabetes to Mitigate Heart Failure Risk: Insights From a Prospective Cohort Study. Diabetes Care. 2024;47(10):1818-1825.
4. Wang X, Ma H, Li X, Heianza Y, Fonseca V, Qi L. Joint association of loneliness and traditional risk factor control and incident cardiovascular disease in diabetes patients. Eur Heart J. 2023;44(28):2583-2591.
5. Li S, Liu Z, Joseph P, et al. Modifiable risk factors associated with cardiovascular disease and mortality in China: a PURE substudy. Eur Heart J. 2022;43(30):2852-2863.
6. Costantino S, Paneni F, Cosentino F. Ageing, metabolism and cardiovascular disease. J Physiol. 2015;594(8):2061-2073.
7. Lloyd-Jones DM, Larson MG, Beiser A, Levy D. Lifetime risk of developing coronary heart disease. Lancet. 1999;353(9147):89-92.
8. Li T, Zhao Y, Yang X, et al. Association between insulin-like growth factor-1 and cardiovascular events: a systematic review and dose-response meta-analysis of cohort studies. J Endocrinol Invest. 2022;45(12):2221-2231.
